# Stat5 Exerts Distinct, Vital Functions in the Cytoplasm and Nucleus of Bcr-Abl^+^ K562 and Jak2(V617F)^+^ HEL Leukemia Cells

**DOI:** 10.3390/cancers7010503

**Published:** 2015-03-19

**Authors:** Axel Weber, Corina Borghouts, Christian Brendel, Richard Moriggl, Natalia Delis, Boris Brill, Vida Vafaizadeh, Bernd Groner

**Affiliations:** 1 Georg-Speyer-Haus, Institute for Tumor Biology and Experimental Therapy, Frankfurt am Main 60596, Germany; E-Mails: axel.weber01@t-online.de (A.W.); delis@gsh.uni-frankfurt.de (N.D.); brill@gsh.uni-frankfurt.de (B.B.); vida.vafaizadeh@unibas.ch (V.V.); 2 Ganymed Pharmaceuticals AG, Mainz 55131, Germany; E-Mail: c.heinz@ganymed.ag; 3 Boston Children’s Hospital, Division of Hematology/Oncology, Boston, MA 02115, USA; E-Mail: christian.brendel@childrens.harvard.edu; 4 Ludwig Boltzmann Institute for Cancer Research (LBI-CR), Vienna 1090, Austria; E-Mail: richard.moriggl@lbicr.lbg.ac.at

**Keywords:** peptide aptamer (PA), signal transducer and activator of transcription 5 (Stat5), RNA interference (RNAi), protein/lentiviral transduction, chronic myeloid leukemia (CML), human erythroid leukemia (HEL), Bcr-Abl, Jak2(V617F), canonical/non-canonical

## Abstract

Signal transducers and activators of transcription (Stats) play central roles in the conversion of extracellular signals, e.g., cytokines, hormones and growth factors, into tissue and cell type specific gene expression patterns. In normal cells, their signaling potential is strictly limited in extent and duration. The persistent activation of Stat3 or Stat5 is found in many human tumor cells and contributes to their growth and survival. Stat5 activation plays a pivotal role in nearly all hematological malignancies and occurs downstream of oncogenic kinases, e.g., Bcr-Abl in chronic myeloid leukemias (CML) and Jak2(V617F) in other myeloproliferative diseases (MPD). We defined the mechanisms through which Stat5 affects growth and survival of K562 cells, representative of Bcr-Abl positive CML, and HEL cells, representative for Jak2(V617F) positive acute erythroid leukemia. In our experiments we suppressed the protein expression levels of Stat5a and Stat5b through shRNA mediated downregulation and demonstrated the dependence of cell survival on the presence of Stat5. Alternatively, we interfered with the functional capacities of the Stat5 protein through the interaction with a Stat5 specific peptide ligand. This ligand is a Stat5 specific peptide aptamer construct which comprises a 12mer peptide integrated into a modified thioredoxin scaffold, S5-DBD-PA. The peptide sequence specifically recognizes the DNA binding domain (DBD) of Stat5. Complex formation of S5-DBD-PA with Stat5 causes a strong reduction of P-Stat5 in the nuclear fraction of Bcr-Abl-transformed K562 cells and a suppression of Stat5 target genes. Distinct Stat5 mediated survival mechanisms were detected in K562 and Jak2(V617F)-transformed HEL cells. Stat5 is activated in the nuclear and cytosolic compartments of K562 cells and the S5-DBD-PA inhibitor most likely affects the viability of Bcr-Abl^+^ K562 cells through the inhibition of canonical Stat5 induced target gene transcription. In HEL cells, Stat5 is predominantly present in the cytoplasm and the survival of the Jak2(V617F)^+^ HEL cells is impeded through the inhibition of the cytoplasmic functions of Stat5.

## 1. Introduction

The Jak/Stat signaling pathway, based on the Janus kinases and the signal transducers and activators of transcription factors, provides an essential cellular communication route from the plasma membrane to the nucleus. It allows extracellular signaling ligands, mainly cytokines, to activate cellular surface receptors and confer their activities onto nuclear transcription patterns. The Stat factors play central roles in the regulation of cell growth, differentiation and metabolism in many different cell types and organs [1]. The regulation of cellular survival and proliferation by Stat3 and Stat5 implies that their inappropriate activation can become an important contributor to cancer [2]. Although Stat3 and Stat5 are structurally similar, they have distinct effects on gene expression and cellular phenotypes [3], but both factors have been shown to act as oncogenes when their activation was deregulated in transgenic animals [4,5].

Under normal conditions, the activation of Stat proteins is transient and tightly controlled. The balance of activating and inactivating components determines the extent and duration of Stat functions [6]. The activating influence of protein tyrosine kinases is balanced by negative regulators, such as SOCS, PIAS and protein tyrosine phosphatase family members [7]. Stat5 initially was identified in mammary epithelium of lactating animals as a latent cytoplasmic transcription factor, which drives the expression of milk proteins in response to the lactogenic hormone prolactin [8,9]. In the meantime we have learned that Stat5a and Stat5b, two closely related isoforms of the molecule [10], convey signals of a plethora of activating ligands into transcriptional programs and cellular phenotypes specific for many individual tissues and their stages of differentiation [11].

The canonical view of Stat activation comprises the phosphorylation of particular tyrosine residues, tyrosine 694 in Stat5a and tyrosine 699 in Stat5b. Phosphorylation can be catalyzed by receptor associated Janus family kinases (Jak), receptor tyrosine kinases (e.g., EGFR) or cytoplasmic kinases (e.g., Src). The reciprocal interaction between the SH2- domain of one Stat molecule and the phosphorylated tyrosine of a second Stat molecule causes the formation of dimers [12]. Parallel dimer formation leads to the exposure of a NLS-signal within the DBD-domain surface. This mediates the nuclear translocation by the importin-α transport system [13,14]. In the nucleus, the DBD-domain recognizes consensus GAS-motifs (TTC(N)_2-4_GAA, gamma interferon activated sites) within target gene promoters, the initial event of gene regulation. Tissue specific gene expression patterns result from the accessibility of genes due to particular chromatin conformations, secondary modifications of gene associated proteins, cofactor-interactions and concomitant epigenetic marking, and gene activating and repressive functions have been described [15,16,17]. Stat5 is a versatile regulator of differentiation specific functions in multiple organs. It contributes to the regulation of homeostasis and organ function in the mammary gland, prostate and liver [5,18,19,20,21]. In addition, Stat5 is a master regulator of hematopoiesis and immune cell functions. Stat5 regulates the self-renewal and expansion of hematopoietic stem and progenitor cells, the differentiation towards committed myeloid and lymphoid blood cell lineages [22,23,24] and is essential for the regulation of the immune response through NK cells, cytotoxic T-cells, regulatory/suppressor or helper T-cells, mast cells, platelets/megakaryocytes and macrophages [25,26,27].

Its influence on cellular proliferation, survival and differentiation is most likely the reason that deregulated activation of Stat5 assumes a role in the pathogenesis of many forms of leukemias and lymphomas [28]. Persistently active Stat5 has been detected in e.g., chronic myelogenous leukemia (CML), acute myelogenous leukemia (AML), acute lymphocytic leukemia (ALL) [29,30] and in Hodgkin lymphomas [31]. Stat5 is induced in leukemias and lymphomas through the action of mutated tyrosine kinases, e.g., Bcr-Abl and Jak2V617F [32,33]. It can also be activated through autocrine or paracrine functions of cytokines. The Bcr-Abl oncoprotein is the driver mutation for chronic myelogenous leukemia (CML), and the Jak2(V617F) point mutation is strongly associated with acute myeloid leukemias (AML) and myeloproliferative diseases (MPD’s) [33,34,35].

The extent of Stat5 activation correlates with the progression of these malignancies [36,37,38]. Specific tyrosine kinases inhibitors (TKI) have been developed for the treatment of leukemias, initially CML, but Stat5 activation has been found to be associated with acquired drug resistance [39]. The inhibition of individual kinases seems insufficient, since Stat5 can be activated by various members of the tyrosine kinase families [35,40]. Alternatively, through the depletion of Stat5 expression drug resistance can be overcome and Bcr-Abl^+^ and Jak2(V617F)^+^ MPNs can be eliminated [33,37]. Stat5 therefore seems a promising therapeutic target and agents which directly interfere with Stat5 mediated functions could become beneficial [29,41,42].

**Figure 1:**
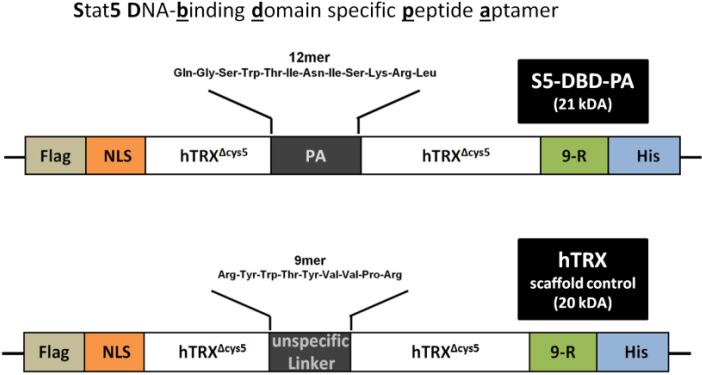
Design of the Stat5 specific peptide aptamer (PA) construct, S5-DBD-PA, a ligand of the DNA-binding domain of Stat5. The recombinant S5-DBD-PA protein comprise functional domains for its purification and transduction into target cells. The 12mer PA sequence, a specific interactor of the DNA-binding domain (DBD) of Stat5, is integrated in the active loop of the optimized hTRX^Δcys5^ scaffold protein, this provides for a constrained conformation of the peptide favorable for target binding and stability. A domain of 9 sequential L-arginine residues (9-R) serves as a protein transduction domain (PTD) and a histidine tag (His) for purification by nickel-affinity chromatography. These domains are present at the C-terminus of hTRX^Δcys5^. An N-terminal Flag epitope provides for the immunodetection of the protein and a NLS-sequence allows for enhanced nuclear import. The S5-DBD-PA construct encodes a protein of 21 kDa. The replacement of the 12mer PA sequence by an unspecific 9mer linker sequence yielded a scaffold control protein construct (hTRX, 20 kDa).

Transcription factors are non-conventional drug targets. They lack hydrophobic binding pockets for drug like compounds which are normally present in enzymatic domains. This complicates the identification of low molecular weight compounds as inhibitors. Protein therapeutics, designed to specifically interfere with functional domains of Stat5 could possibly serve as an alternative. Protein-protein interaction interfaces can be large and flat and do not necessarily include hydrophobic pocket structures [43]. We recently described a peptide based ligand able to bind with high affinity to the DNA-binding domain (DBD) of Stat5 [44]. This ligand is a protein construct comprising a target specific 12mer peptide aptamer (PA) sequence, a protein scaffold (human thioredoxin, hTRX) and functional tags for cellular delivery, protein purification and the detection of S5-DBD-PA (Stat5 DNA-binding domain specific peptide aptamer; Figure 1). Stable S5-DBD-PA monomers were purified after recombinant expression in bacteria [45]. A protein transduction domain (PTD), consisting of nine arginine residues (9-R), allowed us to introduce the recombinant protein into target cells and avoid its intracellular, endosomal degradation.

Reporter gene expression assays were used to show that the introduction into the intracellular milieu resulted in the binding of S5-DBD-PA to the DNA-binding domain of Stat5 and caused a strong inhibition of Stat5 transactivation [44]. The interaction of S5-DBD-PA with the DBD of Stat5 impedes the recognition and binding of Stat5 to GAS DNA-elements. We also showed that S5-DBD-PA causes a reduction of growth and viability of human prostate and breast cancer cells.

We extended our studies of the effects of S5-DBD-PA mediated Stat5 inhibition to leukemic cells and compared the influences of Stat5 inhibition in Bcr-Abl expressing human K562 CML cells and in human erythroid leukemia HEL cells, positive for the Jak2(V617F) point mutation. Two experimental approaches, downregulation of Stat5 with specific shRNAs and S5-DBD-PA mediated Stat5 inhibition were investigated. shRNA mediated downregulation of Stat5 confirmed the essential functions of this transcription factor for the viability of leukemic cells. Apoptosis was induced in K562 and in HEL cells upon depletion of Stat5. In contrast, the intracellular presence of S5-DBD-PA, either upon the endogenous expression of S5-DBD-PA encoded by a viral gene transfer vector or upon protein transduction of the purified S5-DBD-PA recombinant protein, only affected the growth and viability of K562 cells, but not of HEL cells. Our data support the notion that Stat5 exerts distinct nuclear and cytosolic functions in leukemia cells. Different Stat5 mediated survival mechanisms, depending on the presence of nuclear and cytoplasmic Stat5 are active in Bcr-Abl transformed CML and in Jak2(V617F) transformed AML cells. These observations might become important for the choice of specific, leukemia-type dependent drugs in the treatment of cancer patients.

## 2. Results and Discussion

### 2.1. shRNA-Mediated Downregulation of Stat5 Causes Cell Death of Bcr-Abl Positive K562 and of Jak2(V617F) Positive HEL Leukemic Cells

Stat5 signaling is a key contributor to transformation in leukemic cells and is required for the maintenance of leukemic phenotypes [28,33,46]. Stat5 is a substrate of leukemia associated oncogenic kinases, e.g., Bcr-Abl, Flt3-ITD, c-Kit(D816V), Npm-Alk or Jak2(V617F) and promotes cellular growth and survival [36,37,47,48]. Since both, Stat3 and Stat5 can assume oncogenic functions, we investigated the activation patterns of both Stat family members in human CML and AML cell lines (Figure S1a). Stat5 was activated in all cell lines examined, with the exception of the Kasumi-1 cells, whereas Stat3 activation was only observed in HEL cells.

A similar analysis was carried out with IL-3-dependent murine hematopoietic Ba/F3 cells and derivatives of these cells expressing leukemia associated oncogenic kinases (Figure S1b). The individual cell lines expressed oncoproteins with activating mutations: Stat5, cS5^f^: S710F, c-Kit, caKit: D816V, Bcr-Abl, Tel-Jak and Npm-Alk. All of these lines grow IL-3 independently due to the activation of Stat5, but not of Stat3 [49,50,51,52,53]. Exceptions are the Tel-Jak2 and Jak2(V617F) expressing cell lines which also show enhanced Stat3 activation.

**Figure 2:**
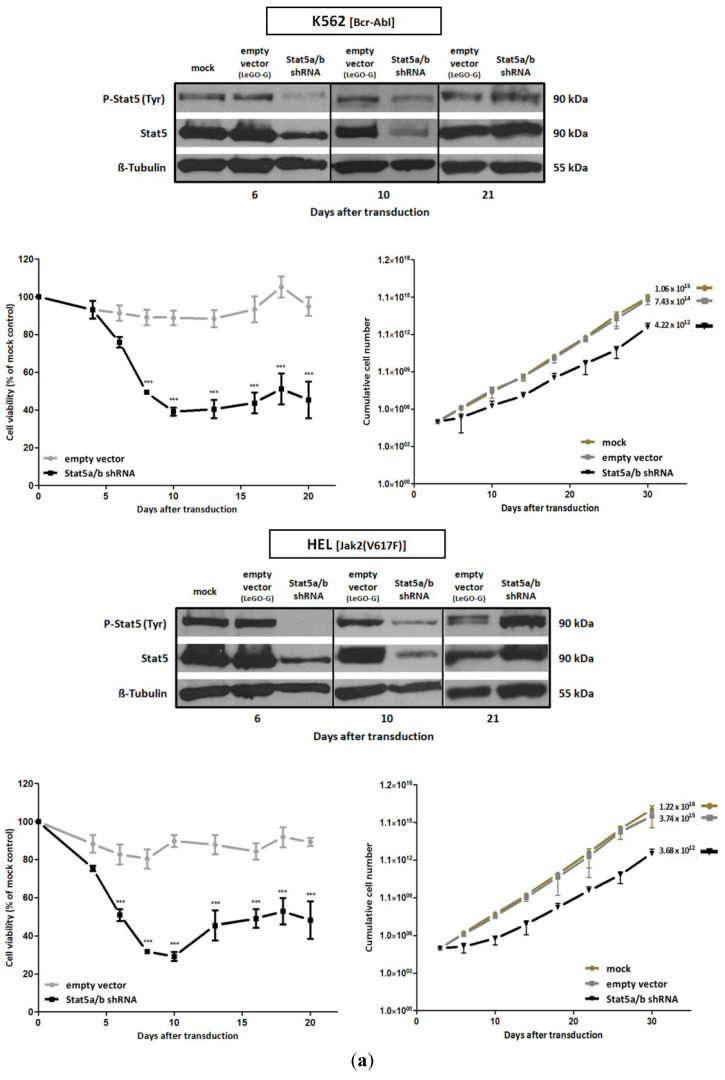

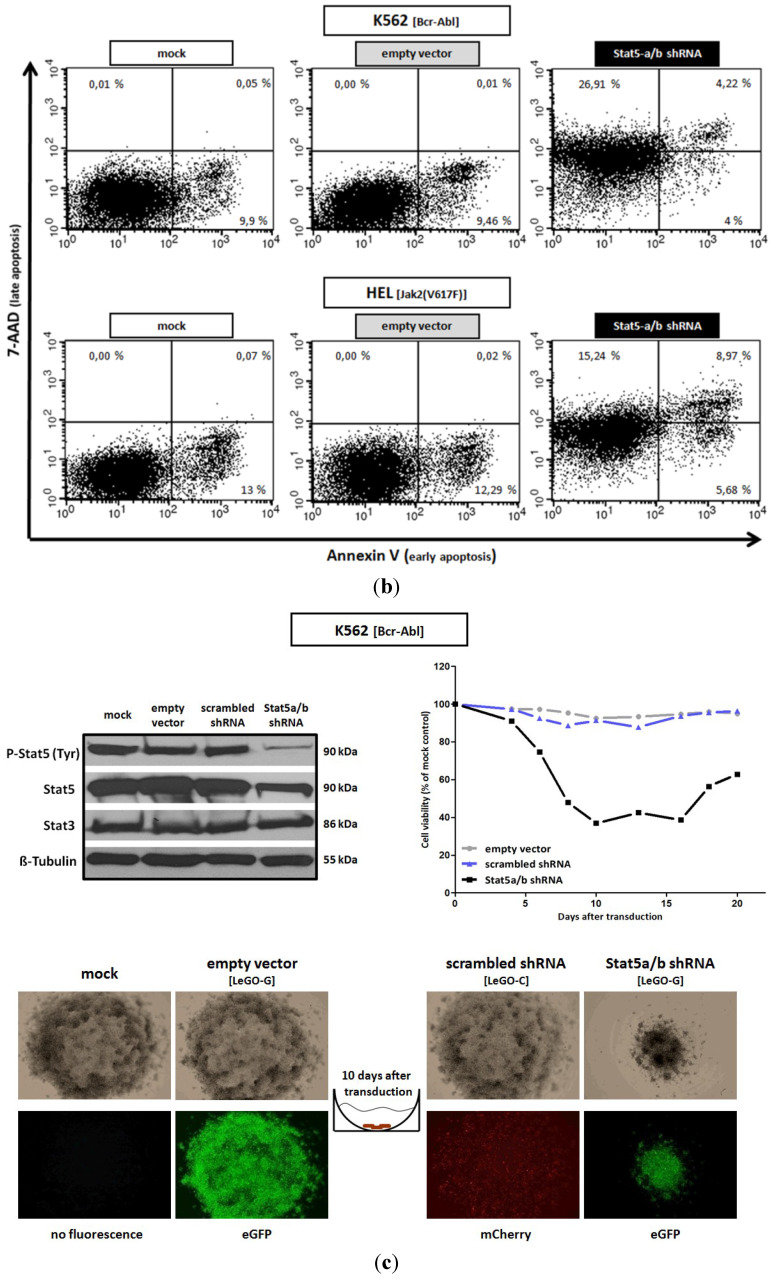

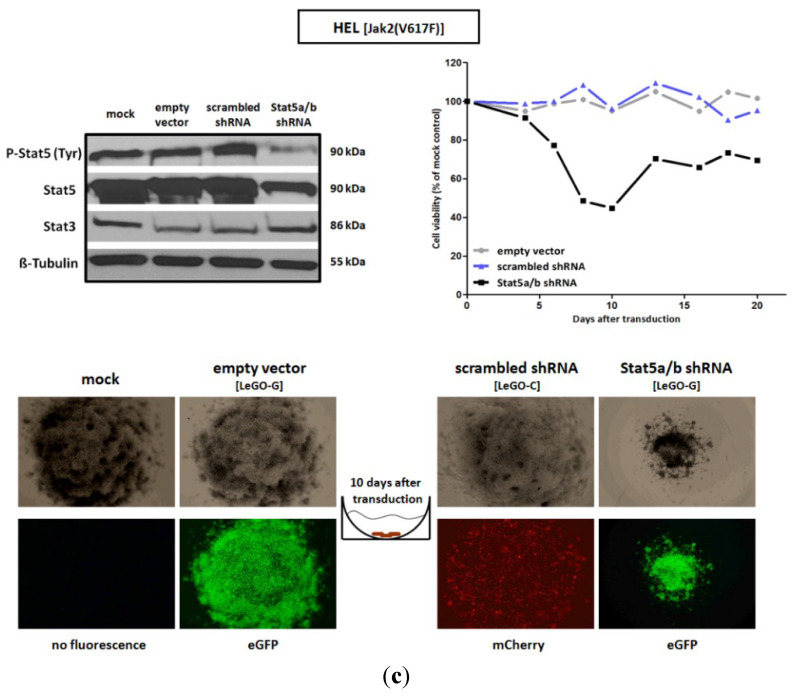
shRNA-mediated downregulation of Stat5 expression strongly reduces the survival of Bcr-Abl expressing K562 and Jak2(V617F) expressing HEL cells. (**a**) shRNA mediated downregulation of Stat5a and Stat5b expression. K562 and HEL cells were transduced with lentiviral vectors encoding shRNA directed against both Stat5 isoforms. Mock treated and empty vector (LeGO-G) transduced cells served as controls. Western blot analyses was used to visualize the expression of Stat5 and activated P-Stat5 6, 10 and 21 days after infection of the cells. Cell proliferation and viability were assayed over a period of 20 days after infection by XTT measurement (*n* = 4; Ø ± SD). Significantly reduced XTT-values (percentage of mock control) were found when the cells were compared to empty vector expressing cells *** *p* < 0.001 (2-way-ANOVA with Bonferroni correction). Growth analyses were carried out by counting the cumulative cell numbers at each passage from day 3 to day 30 after infection (*n* = 3; Ø ± SD); (**b**) Apoptosis measurement by Annexin V/7-AAD staining. Cells were stained and analyzed 10 days after transduction with shRNA-encoding lentiviral vectors. Divided FACS dot plots indicate unstained vital cells (lower left), early apoptotic cells positive for Annexin V (lower right), Annexin V/7-AAD double positive apoptotic cells (upper right) and late apoptotic/necrotic cells positive for -AAD (upper left); (**c**) In a control experiment K562 and HEL cells were treated with a lentiviral vector (LeGO-C) expressing a scrambled shRNA. Cell viability was measured over a period of 20 days by XTT conversion, whereas the corresponding suspension cell mass was documented after 10 days in assay-round bottom wells by phase contrast and fluorescence microscopy. After 14 days cell lysates were analyzed by western blotting with antibodies detecting Stat5 or P-Stat5, detection of Stat3 served as a control for the specificity of the of shRNA.

We investigated the dependence of Bcr-Abl positive K562 cells and Jak2(V617F) positive HEL cells on the presence of Stat5. For this purpose we downregulated the expression of Stat5 in human K562 and HEL cells infected with lentiviral gene transfer vectors encoding shRNA specific for Stat5a and Stat5b. The downregulation of Stat5 strongly affected the viability of the Bcr-Abl^+^ K562 cells (Figure 2a) confirming earlier observations [54,55,56]. K562 cell proliferation was suppressed for 3 weeks. After that time, Stat5 expression and cellular growth rates resumed at normal levels. Similar effects were observed in Jak2(V617F) expressing acute erythroid leukemic HEL cells. We investigated the mechanisms by which the loss of viability and the resumption of cell growth after three weeks could be explained. They could possibly be due to the loss of Stat5 deficient cells and the subsequent outgrowth of a small cell population which had escaped the infection by the shRNA encoding viral vector or had silenced the integrated proviral gene. Since the lentiviral vector also encodes the eGFP marker gene, we followed its expression as a function of time after the viral infection. We observed that the fraction of eGFP expressing cells decreased successively from about 90% to 20% over a period of nine weeks. The fluorescence intensity of the cells, however, decreased much more rapidly within the first three weeks after infection (Figure S3). We conclude that shortly after the infection with the Stat5-shRNA encoding virus, the majority of the K562 and HEL cells die. The resumption of cell growth after prolonged periods could then be due to a minority of uninfected cells or cells in which the U6 promoter driving the shRNA expression had been silenced through epigenetic modifications. The infected cells die within 3 to 5 days. The staining of infected cells with Annexin V/7-AAD confirmed the apoptosis induction by the shRNA mediated downregulation of Stat5 (Figure 2b). The infection of the cells with a viral vector encoding a scrambled shRNA sequence served as a control. K562 and HEL cell growth and viability was not affected (Figure 2c).

The cytotoxic effects of Stat5 downregulation was confirmed in a second CML line expressing the Bcr-Abl fusion protein. Ku812 leukemia cells showed reduced cellular growth and viability shortly after virus infection encoding Stat5-shRNA (Figures S2 and S3). The AML cell line, Kasumi-1, also responded to the expression of Stat5-shRNA. These acute myeloblastic leukemia cells express the oncogenic and leukemia associated AML1-ETO fusion protein, lower levels of the Stat5 protein then other human CML and AML cell lines and contain no activated P-Stat5 (Figure S1a). The influence of Stat5 downregulation on the viability and growth of these cells is therefore not necessarily expected. We suggest that interference with non-canonical functions of Stat5, different from target gene transactivation, might be responsible for it. Such functions have been postulated for latent and activated Stat3 and Stat5 and might involve cofactor activities, the maintenance of heterochromatin, the cytoskeleton and cell organelle structure and functions [1,57,58,59]. Similar observations were made with breast cancer cells. We previously showed that Stat5-shRNA strongly affects the growth and viability of T-47D breast cancer cells in the absence of Stat5 activating stimuli [44].

**Figure 3:**
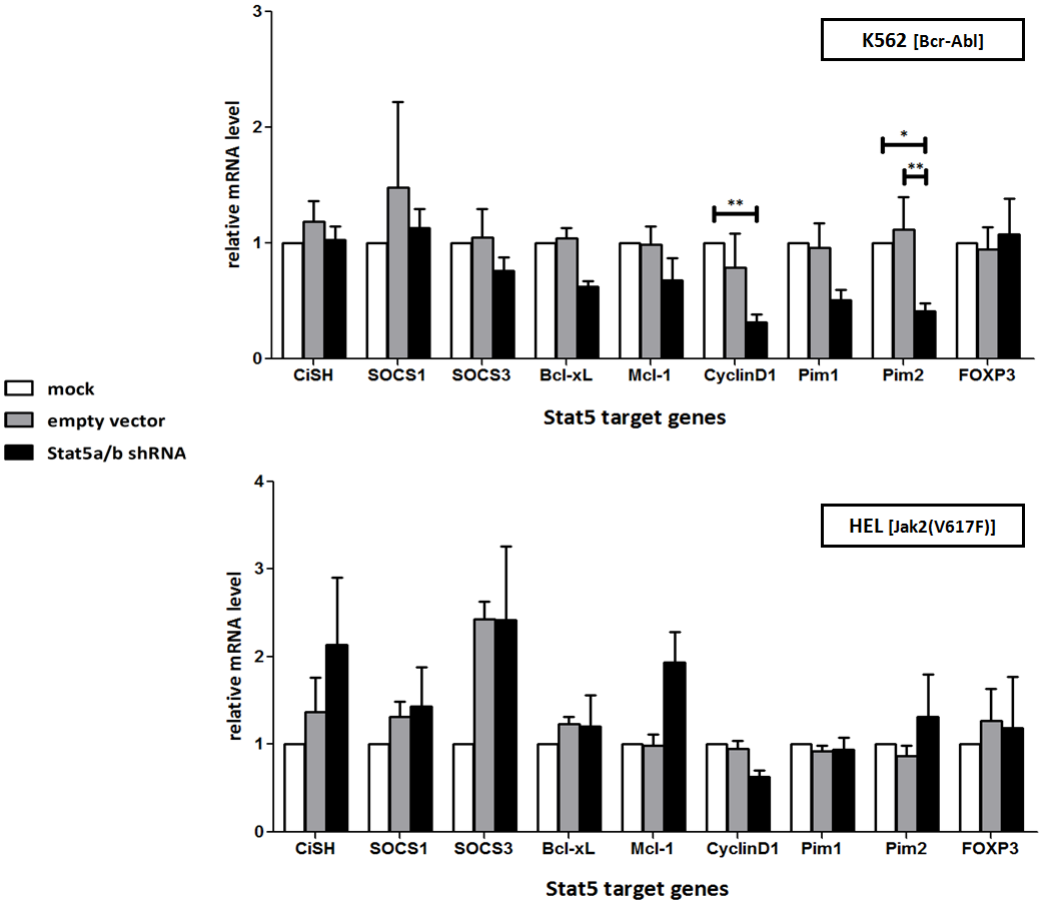
Stat5 specific shRNA reduces the expression of Stat5 target genes Bcl-xL, Cyclin D1 and Pim2 in Bcr-Abl^+^ K562, but not in Jak2(V617F)^+^ HEL cells. Relative expression level of selected Stat5 target genes were analyzed by qRT-PCR in lentivirus transduced K562 and HEL cells. The cells were lysed after seven days and total RNA was extracted for qRT-PCR measurement. Data were normalized to HPRT1 housekeeping gene expression and the relative levels are shown as folds of mock treated control cells (*n* = 3; Ø ± SD). Significantly reduced gene expression level of shRNA expressing cells in comparison to mock and empty vector expressing control cells are indicated. * *p* < 0.05, ** *p* < 0.01 (2-way-ANOVA with Bonferroni correction).

Activated Stat5 is constantly replenished in K652 cells by Bcr-Abl and in HEL cells by Jak2(V617F) activities. It seems reasonable to assume that the effects of Stat5-shRNA transduction in K562 and HEL cells are predominantly resulting from the inhibition of Stat5-regulated transcription and the expression of the Stat5 targets, e.g., d-type cyclins, Bcl-2 family members and Pim genes (serine/threonine protein kinases), crucial for the survival of normal hematopoietic and leukemic cells [60,61,62,63,64]. We measured the expression of these genes as a function of Stat5 downregulation, but only in K562 cells significant reductions were detectable, in accordance with the observed growth suppressing effect of the Stat5-shRNA (Figure 3).

Different disease phenotypes are caused by the Bcr-Abl and Jak2(V617F) tyrosine kinase activities in leukemic cells and this might reflect variable downstream signaling events and transcriptional regulation [65,66,67,68]. We investigated the influence of Stat5 downregulation on the expression of recognized Stat5 target genes (Figure 3). The T-cell regulatory transcription factor FOXP3 and members of the SOCS gene family (CiSH, SOCS1, SOCS3) are components responsible for the negative feedback regulation of cytokine induced Jak/Stat signaling [60,69]. The expression of FOXP3 was not influenced by Stat5 downregulation in K562 cells. The same was true for SOCS1 and SOCS3 expression. Unexpectedly, HEL cells showed an increase in SOCS expression after infection with the Stat5-shRNA encoding lentivirus. This could possibly be explained by the release of interferons (IFN) and the activation of other Stat proteins, e.g., Stat3 and Stat1, upon viral infection. Jak2(V617F) kinase activity in HEL cells could possibly cause the phosphorylation of other Stat family members, which could compensate for the loss of Stat5 function. Gene expression profiling studies in ET-patients revealed that the absence of mutant Jak2(V617F) correlates with lower P-Stat3 levels and the reduced expression of target genes like Pim-1 and SOCS-2 [70]. Alternatively, the relief of negative regulation exerted by Stat5 could play a role [71].

### 2.2. Protein Transduction of the Recombinant Stat5 Specific Ligand S5-DBD-PA and Intracellular Expression of a S5-DBD-PA Encoding gene Transfer Vector in K562 and HEL Cells

**Figure 4:**
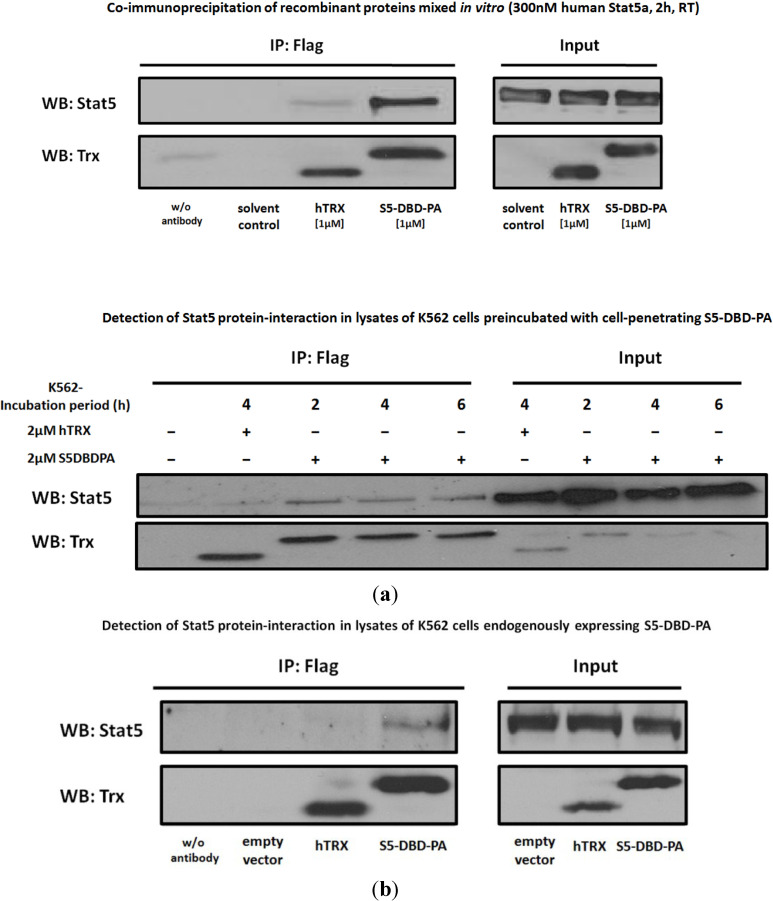

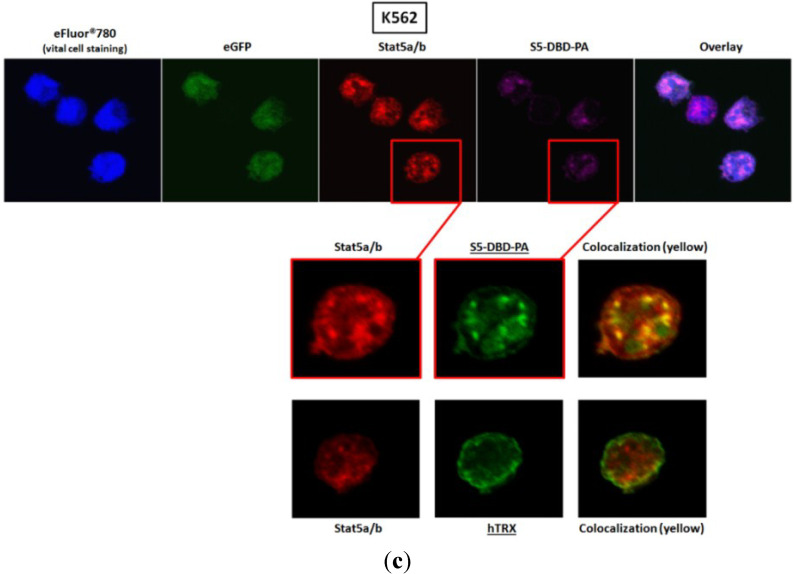
S5-DBD-PA specifically interacts with Stat5 and intracellularly colocalizes with Stat5. (**a**) Co-immunoprecipitation (Co-IP) of recombinant S5-DBD-PA and Stat5. Co-IP studies were carried out either with a mixture of recombinant S5-DBD-PA (f.c.: 1 µM) and recombinant human Stat5a protein (f.c.: 300 nM), incubated for 2 h at room temperature (RT), or with lysates of K562 cells incubated for 2, 4 and 6 h with S5-DBD-PA (f.c.: 2 µM). S5-DBD-PA was precipitated with a Flag-tag antibody. Immunoprecipitates were examined by Western blotting with thioredoxin and Stat5 specific antibodies. The use of non-antibody loaded beads as well as the incubation with protein solvent (dialysis buffer) and with the non-specific scaffold control protein hTRX (f.c.: 1 or 2 µM) served as controls. During the time of S5-DBD-PA addition to K562 cells, the cells were cultured in starvation medium (2% FCS). Spuriously attached proteins were removed by acid-wash prior to lysate preparation. Input represents 10% of the sample volume used for the Co-IP experiment; (**b**) Co-IP analysis of the S5-DBD-PA/Stat5 interaction after endogenous expression of S5-DBD-PA in target cells. K562 cells were infected with lentivirus encoding either the empty vector (SiEW) or encoding the S5-DBD-PA and hTRX proteins. Lysates were prepared seven days after infection and used for IP with a Flag-tag antibody. Protein constructs and co-precipitated Stat5 were subsequently analyzed by western blotting with Stat5 or thioredoxin specific antibodies. Unspecific binding was controlled by lysate incubation with beads without antibodies. Input represents 10% of the lysate volume used for the Co-IP experiment; (**c**) Confocal immunofluorescence microscopy of K562 cells endogenously expressing S5-DBD-PA. Images were taken seven days after transduction with a lentiviral S5-DBD-PA gene transfer vector. Cells were stained with a Flag-tag and a Stat5 antibody either marked with a Alexa^®^546 or Alexa^®^647 conjugated secondary antibody. Viable cell staining was performed with eFluor^®^780 and fluorescence marker (eGFP) expression of the SiEW lentiviral vector was monitored. Protein colocalization was visualized by red/green image merging. The staining of hTRX expressing K562 control cells is shown on the bottom.

We analyzed the binding of S5-DBD-PA to Stat5 in K562 cells by antibody co-immunoprecipitation experiments and by immunofluorescence co-localisation experiments. We initially confirmed the ability of S5-DBD-PA to interact with Stat5 in a cell free protein lysate in a control experiment. When S5-DBD-PA was added to a cellular lysate of K562 cells and protein complexes were immunoprecipitated with a flag specific antibody, the presence of Stat5 could be demonstrated in the immunoprecipitate upon western blotting (Figure 4a). Subsequently, S5-DBD-PA was either transduced as a recombinant protein into K562 cells or its intracellular expression was induced upon infection of the cells with a S5-DBD-PA encoding viral vector. In the protein transduction experiments, K562 cells were treated for 2, 4 and 6 h with S5-DBD-PA or the control protein hTRX, *i.e.*, the scaffold protein lacking the PA ligand domain. The results show that Stat5 was co-precipitated with S5-DBD-PA (Figure 4a) and suggest that the transduced S5-DBD-PA forms an intracellular complex with its target protein. This experiment also indicates that the intracellular S5-DBD-PA, taken up by the cells is able to escape endosomal and lysosomal degradation.

These results were corroborated by a complementary experimental approach. S5-DBD-PA was endogenously expressed in K562 cells upon infection with a S5-DBD-PA encoding lentiviral gene transfer vector. Again, co-immunoprecipitation experiments and western blotting analysis showed that the intracellularly expressed S5-DBD-PA and Stat5 are present in a protein complex (Figure 4b). Confocal immunofluorescence microscopy was used to visualize the intracellular co-localization of S5-DBD-PA and Stat5 (Figure 4c).

### 2.3. Activated Stat5 Is Present in the Nucleus of K652 Cells, but Nearly Absent from the Nucleus of HEL Cells; S5-DBD-PA Interferes with Nuclear Translocation, Target Gene Transactivation and Reduces the Level of the Transcription Factor in K562 and HEL Cells

**Figure 5:**
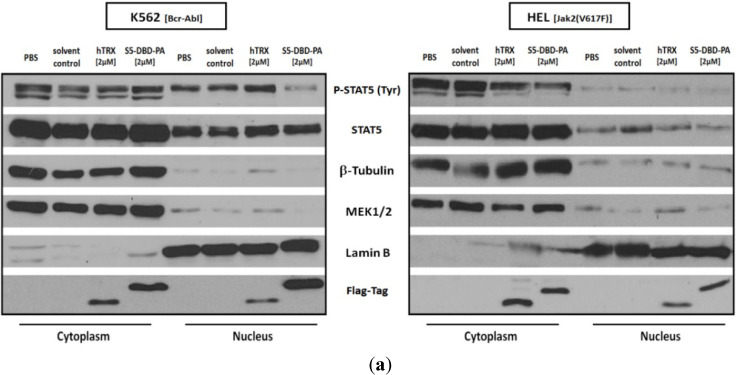

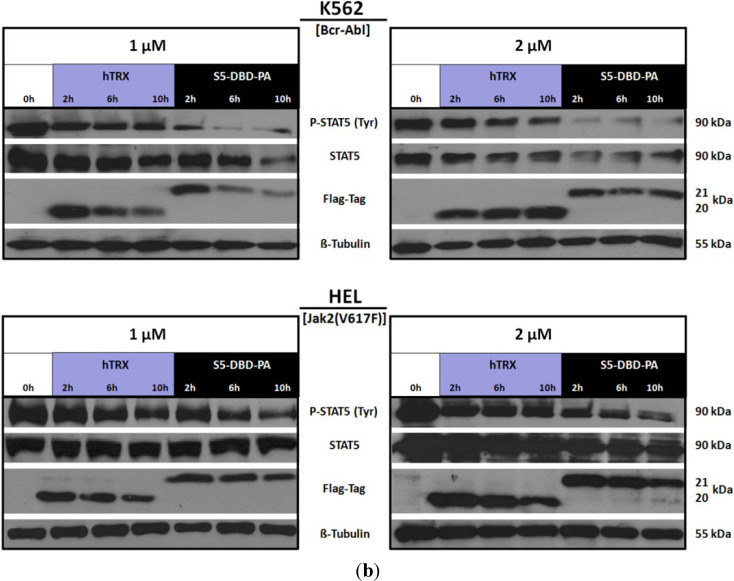
The interaction of S5-DBD-PA with the DBD of Stat5 inhibits nuclear translocation of activated dimers of Stat5 in K562 cells and enhances Stat5 degradation. (**a**) Recombinant S5-DBD-PA and hTRX were added to the culture media of K562 and HEL cells (f.c.: 2 µM). After 4 h incubation, membrane bound proteins were removed by acid-wash and cytosolic and nuclear fractions were prepared. Cell fractions subsequently were analyzed by western blotting with antibodies detecting Flag-tagged recombinant proteins, total Stat5 and tyrosine phosphorylated, P-Stat5. Antibodies recognizing the cytosolic markers MEK1/2 and β-Tubulin and antibodies directed against nuclear Lamin B were used to monitor the quality of the subcellular fractionation. The treatment with PBS or protein solvent served as negative controls; (**b**) Cellular uptake of recombinant S5-DBD-PA by protein transduction. K562 and HEL cells were seeded in culture media supplemented with S5-DBD-PA or the hTRX scaffold control protein (f.c.: 1 and 2 µM). Cells were lysed after 2, 6 and 10 h. Non-transduced, membrane-bound proteins were removed for accurate measurement. Recombinant protein uptake and Stat5 were analyzed with a Flag-tag antibody and antibodies recognizing either Stat5 or P-Stat5.

The analysis of nuclear and cytosolic fractions of K562 and HEL cells yielded interesting insights into the subcellular distribution of latent and activated Stat5 in these cells and into the mechanism of action of S5-DBD-PA (Figure 5a). In the Bcr-Abl positive K562 cells, activated Stat5 is present in the cytoplasm and in the nucleus. An isoform of Stat5 with a slightly lower molecular weight was detected in the cytoplasm. In the Jak2(V617F) positive HEL cells, activated Stat5 is largely restricted to the cytoplasmic fraction. When these cells were treated with S5-DBD-PA or the hTRX control, the recombinant proteins, detected with the flag tag specific antibodies, were present in both subcellular compartments. The intracellular presence of S5-DBD-PA, however, caused a strong reduction of activated Stat5 in the nuclear fraction of K562 cells and in the cytoplasmic fraction of HEL cells. These effects were specific for the S5-DBD-PA ligand and not exerted by the control hTRX protein (Figure 5a). These results are in line with our previous observations [44]. Endogenously expressed S5-DBD-PA impaired the nuclear translocation of prolactin induced Stat5-dimers in breast cancer cells. It is therefore conceivable that S5-DBD-PA also interferes with the nuclear translocation of constitutively activated Stat5 in Bcr-Abl^+^ K562 cells. Lamin B and β tubulin served as nuclear and cytoplasmic markers.

The near exclusive presence of activated Stat5 in the cytoplasmic fraction of HEL cells is not necessarily expected. Interestingly, the activated, cytoplasmic Stat5 is also diminished by the transduction of S5-DBD-PA into these cells (Figure 5a). Cytoplasmic retention of Stat5 has been previously observed in Bcr-abl positive leukemic cells and aberrantly active Src-family kinases and secondary Stat5 protein modifications, e.g., by acetylation or serine phosphorylation, have been implied [72,73]. Our results suggest that cytoplasmic retention of activated Stat5 also occurs in acute leukemia cells, triggered by the Jak2(V617F) kinase. A cytoplasmic function of Stat5 in leukemic cells could be linked to its influence on PI3K/Akt/mTOR signaling and the regulation of leukemic cell survival functions [74,75,76,77]. Direct interactions of the p85 regulatory subunit of PI3-kinase and the Gab2 adaptor protein with P-Stat5 form an activating complex in the cytoplasm and promote downstream Akt signaling [48]. This cytoplasmic function of Stat5 could also be active in HEL cells enhancing the PI3K-mediated induction of the MAPK pathway [78]. The cytoplasmic cofactor activities seem to be indispensable for HEL cells, a conclusion based on the cell death inducing effect of the Stat5-shRNA.

We treated K562 cells and HEL cells with 1 or 2 µM S5-DBD-PA for increasing lengths of time and confirmed the observation that phosphorylated Stat5 is being diminished upon S5-DBD-PA transduction (Figure 5b). The extent of P-Stat5 reduction, however, differed in the two cell lines. A more pronounced effect was observed in K562 cells. Since S5-DBD-PA interacts with a Stat5 domain distant from its phosphorylation site, this effect is not necessarily expected. S5-DBD-PA shares similarities with a previously investigated construct, rS3-PA, which we used to interrogate functions of Stat3. This protein interacts with the dimerization domain of Stat3, adjacent to the tyrosine phosphorylation site and also resulted in a reduction of phosphorylated Stat3 [79]. It exerts its effect through masking the access of the kinase to its substrate. In the case of S5-DBD-PA, we suspect that the complex formation of S5-DBD-PA with the DBD of Stat5 enhances the degradation of P-Stat5, a process more pronounced in the nucleus of K562 cells when compared to the cytoplasm of HEL cells. This might be reflected in the distinguishable functions of Stat5 in these cells. K562 cells seem dependent on Stat5 regulated gene expression, whereas HEL cells require the cytoplasmic Stat5 activities. The S5-DBD-PA Stat5 complex seems less susceptible for degradation in HEL cells and the cytoplasmic activities of Stat5 seem less strongly affected by the S5-DBD-PA interaction. Although S5-DBD-PA does not directly interact with the sequences immediately surrounding the tyrosine phosphorylation site of Stat5, we cannot exclude that the recombinant protein of 21 kDa could also interfere with the access of Bcr-Abl to its substrate and thus hamper the persistent phosphorylation of Stat5.

**Figure 6:**
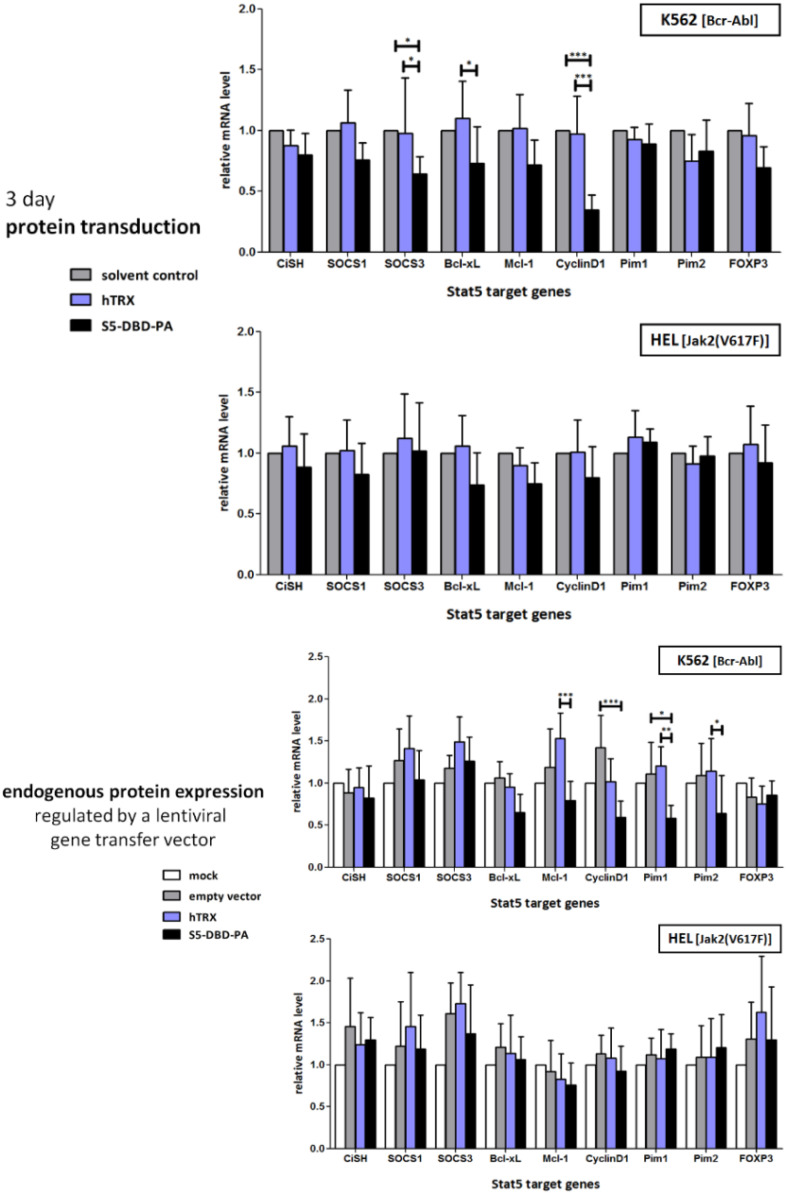
S5-DBD-PA interferes with Stat5 target gene transactivation in Bcr-Abl^+^ K562 CML-cells, but not in Jak2(V617)^+^ HEL cells. mRNA expression of selected Stat5 target genes by qRT-PCR measurements. S5-DBD-PA or hTRX scaffold control proteins were delivered into K562 and HEL cells either by protein transduction or lentiviral gene transfer. For analyzing the influence of protein transduction, cells were cultured under normal conditions and incubated for 3 days with the recombinant protein constructs. During this time S5-DBD-PA (f.c.: 1 µM), hTRX (f.c.: 1 µM) or the same volume of solvent control were added 4 times to the culture media (after 0, 24, 48 and 66 h). In a separate experiment both cell lines were infected with lentiviruses, either encoding S5-DBD-PA, hTRX or the empty vector (SiEW) and analyzed after 10 days. Cells were lyzed and total RNA was extracted for qRT-PCR measurement. Data were normalized to HPRT1 housekeeping gene expression and relative expression levels were depicted either as folds of protein solvent or mock treated control cells (*n* = 5; Ø ± SD). Significantly reduced gene expression level of S5-DBD-PA treated cells in comparison to hTRX, protein solvent or empty vector treatment are indicated. * *p* < 0.05, ** *p* < 0.01, *** *p* < 0.001 (2-way-ANOVA with Bonferroni correction).

The preferential presence of P-Stat5 in the nucleus of K562 cells and in the cytoplasm of HEL cells suggests that the activated transcription factor might exert different effects on the expression of Stat5 target genes in these two cell lines. We measured the extent of expression of suspected Stat5 target genes in K562 and HEL cells and compared the effects of Stat5 inhibition through S5-DBD-PA on the expression levels. Again, we applied two methods to achieve the intracellular presence of S5-DBD-PA, incubation of the cells with the membrane penetrating recombinant S5-DBD-PA protein and infection of the cells with the S5-DBD-PA encoding gene transfer vector. Both approaches caused the reduction of expression levels of Stat5 regulated genes (Figure 6). The extent of the reduction in Stat5 target gene expression was much more pronounced in K562 cells when compared to that in HEL cells.

The treatment of the cells with the recombinant protein also reduced the expression of SOCS1 and SOCS3, an effect not observed in cells infected with the viral S5-DBD-PA expression vector. It is possible that the viral infection caused the induction of IFN, subsequently Stat1 and Stat3 activation and SOCS expression. Similar effects on the expression of survival regulating genes were previously observed upon treatment of K562 cells with tyrosine kinase and Stat5 inhibitors [80,81]. The expression of the Pim proto-oncogenes remained unaffected by the treatment of K562 cells with recombinant S5-DBD-PA, but was strongly affected by the virally encoded protein. The extent of inhibition with exogenously supplied 1 µM S5-DBD-PA was probably not sufficient to interfere with the expression of these genes. This is reminiscent of the results observed with Bcr-Abl^+^ Ku812 cells. High concentrations, 5 µM, of the Stat5 inhibitor pimozide were required to cause a significant reduction of Pim-1 gene expression [42].

### 2.4. The Stat5 Inhibitor S5-DBD-PA Strongly Suppresses Growth and Viability in K562 Cells and, to A Lesser Extent, in HEL Cells

**Figure 7:**
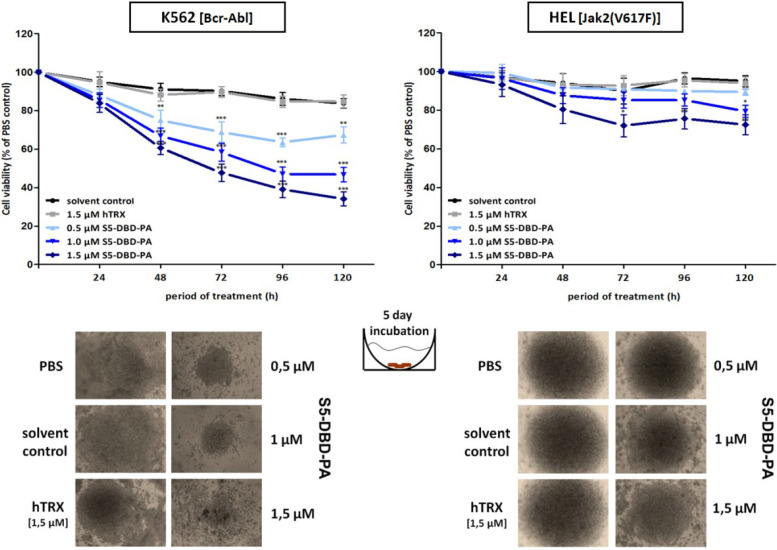
Suppression of K562 and HEL leukemic cell growth and viability by the recombinant cell-penetrating S5-DBD-PA. S5-DBD-PA protein was added to the culture media of K562 and HEL cells in final concentrations of 0.5, 1, 1.5 µM. For negative control 1.5 µM of the non-specific hTRX scaffold protein construct and the same volumes of protein-solvent (dialysis buffer) and PBS were added. Medium and proteins were replaced daily and cell viability and growth were determined by XTT assay over 5 consecutive days. Results are shown as the percentage of viable cells compared to the PBS control (*n* = 4; Ø ± SD). Significantly reduced XTT-values in comparison to protein solvent treated cells are indicated. * *p* < 0.05, ** *p* < 0.01, *** *p* < 0.001 (2-way-ANOVA with Bonferroni correction).

We measured the effects of S5-DBD-PA treatment and its inhibition of Stat5 functions on the survival of K562 and HEL cells. The addition of recombinant S5-DBD-PA to the cell culture media revealed a dose-dependent decrease in the growth and viability of K562 cells. HEL cells were also affected, but to a lesser extent (Figure 7). This response might be caused by the reduction of Stat5 levels, most likely due to the enhanced degradation of Stat5. The reduction of P-Stat5 levels are also the probable cause of the increased sensitivity of these cells to Stat5 and Jak kinase inhibitors, indicating essential roles of the Stat5 mediated cytoplasmic functions [41]. The Stat5 inhibitor pimozide was shown to reduce the viability of Bcr-Abl positive K562 and Ku812 cells [42,81].

**Figure 8:**
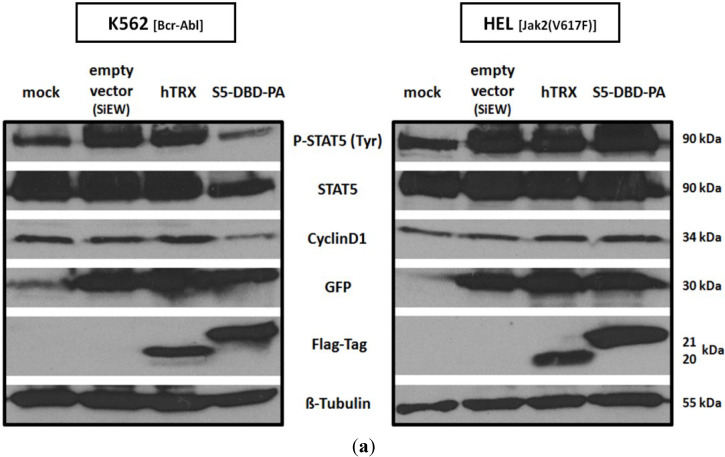

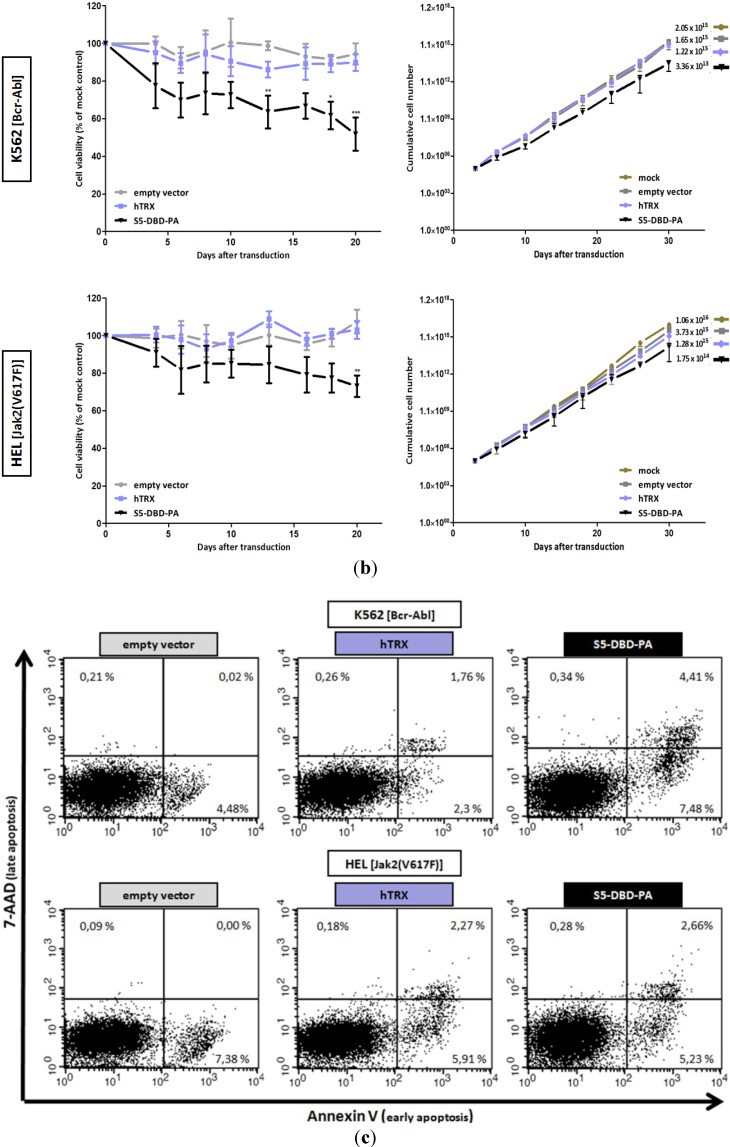

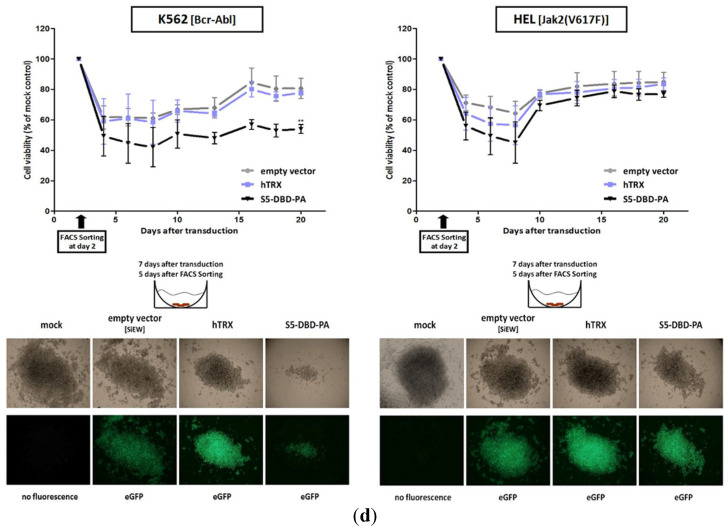
Reduction of K562 and HEL cell survival after infection with the S5-DBD-PA encoding lentivirus. (**a**) Bcr-Abl^+^ K562 and Jak2(V617F)^+^ HEL cells were infected with SiEW lentiviral vectors encoding either S5-DBD-PA, hTRX or an empty vector as controls. After 7 days, cell lysates were prepared and analyzed by western blotting for the expression of the encoded proteins with a Flag-tag antibody. Additional antibodies were used for the detection of Stat5 and P-Stat5 as well as for the analysis of eGFP fluorescence marker and Cyclin D1 target gene expression; (**b**) Proliferation and viability of the cells were monitored with the XTT-assay (*n* = 4; Ø ± SD), cell growth was analyzed by counting the cumulative cell numbers at each passaging interval (*n* = 3; Ø ± SD). Graphs indicate significantly reduced XTT-values (percentage of mock control) in comparison to empty vector expressing cells. * *p* < 0.05, ** *p* < 0.01, *** *p* < 0.001 (2-way-ANOVA with Bonferroni correction); (**c**) Analysis of apoptosis induction by Annexin V/7-AAD staining. 10 days after virus transduction cells were stained and analyzed by FACS. Divided FACS dot plots indicate unstained vital cells (lower left), early apoptotic cells positive for Annexin V (lower right), Annexin V/7-AAD double positive apoptotic cells (upper right) and late apoptotic/necrotic cells positive for 7-AAD (upper left); (**d**) eGFP expressing K562 and HEL cells were FACS sorted 2 days after infection with the lentiviruses and analyzed for changes in viability and growth by XTT conversion. Results are shown as the percentage of viable cells compared to mock control (*n* = 3; Ø ± SD). Significantly reduced XTT-values in comparison to empty vector expressing cells are indicated. ** *p* < 0.01 (2-way-ANOVA with Bonferroni correction). Phase contrast and fluorescence microscopy images of accumulated cells at the round-bottom of assay-96 well plates were taken 7 days after virus transduction (5 days after cell sorting and seeding).

Similar effects were observed in cells in which the expression of the S5-DBD-PA construct was induced by the lentiviral gene expression vector. S5-DBD-PA expression caused the reduction of P-Stat5 and of Cyclin D1 levels in K652 cells (Figure 8a) and a decrease in growth and survival (Figure 8b). Different observations were made in HEL cells. Cyclin D1 levels were not affected by S5-DBD-PA expression and cell viability was reduced to a lesser extent than in K562 cells (Figure 8b). The gene transfer vector, used in our experiments, encodes a eGFP gene in addition to the S5-DBD-PA inhibitor. Infection of K562 and HEL cells with the viral vector initially causes a large fraction of the cells to express the eGFP gene. The co-expression of S5-DBD-PA subsequently leads to Stat5 inhibition and a gradual reduction of eGFP-positive cells over a period of several weeks. The decrease of eGFP fluorescence, when compared to hTRX expressing cells, corroborated the anti-proliferative effect of S5-DBD-PA expression (Figure S6). Again, similar, but less pronounced effects were observed in HEL cells. Apoptosis measurement, 10 days after virus infection, showed an enhanced apoptosis induction in S5-DBD-PA expressing K562 cells, HEL cells were much less affected (Figure 8c). Finally, FACS sorting was carried out with acutely infected cells to gauge and compare the effectiveness of S5-DBD-PA in K562 and HEL cells. High expression of eGFP and of S5-DBD-PA expression is mediated through the common SFFV-promoter. The viability of both cell lines was negatively affected. The control cells and S5-DBD-PA expressing HEL cells recovered after about 10 days, the viability of S5-DBD-PA expressing K562 cells remained significantly reduced (Figure 8d).

Differences in the phenotypic consequences of S5-DBD-PA expression were also observed in Bcr-Abl positive Ku812 and AML1-ETO positive Kasumi-1 cells. Dose-dependent inhibition of cellular viability upon S5-DBD-PA protein transduction was observed (Figure S4). A more pronounced effect was exerted upon endogenous S5-DBD-PA expression. Viability and growth of Ku812 cells were significantly reduced (Figures S5 and S6). The AML cell line, Kasumi-1, showed a weaker response. Kasumi-1 cells grow independently of P-Stat5, but shRNA experiments indicated that the line requires the expression of Stat5 for its survival. The delayed influence of S5-DBD-PA expression on these might be mediated via the inhibition of essential, non-canonical functions of Stat5, similar to the effects observed in HEL cells.

## 3. Experimental Section

### 3.1. Cell Lines and Culture Conditions

The human erythromyeloblastoid CML cell lines K562 (ATCC: CCL-243) and Ku812 (ATCC: CRL-2099) express the p210 subtype of the Bcr-Abl fusion protein. The Jak2(V617F)^+^ human erythroid leukemia cell line HEL (M6 subtype of AML in the French-American-British (FAB) classification; ATCC: TIB-180) is characterized by an erythropoietin independent growth of erythroid progenitors, comparable to the polycythemia vera (PV) disease [82]. Human Kasumi-1 cells are positive for the t(8;21) translocation and express the AML1-ETO fusion protein (M6 subtype of AML in the French-American-British (FAB) classification; ATCC: CRL-2724). All leukemic cell lines were cultured in RPMI 1640 medium (20% FCS). Wild type murine Ba/F3 B-cell progenitor cells were supplemented with 1 ng/mL IL-3 for cultivation. Transformed Ba/F3 variants grow IL-3 independent and were obtained from the Ludwig-Boltzmann Institute Vienna (group of Dr. Richard Moriggl). The lentiviral producer cell line 293T (HEK-293T, ATCC: CRL-11268) was grown in DMEM medium (10% FCS). Media were obtained by Gibco^®^ Life Technolgies (Carlsbad, CA, USA) or Lonza Group (Basel, Switzerland) and supplemented with FCS, L-glutamine (2 mM) and penicillin/streptomycin (100 U/mL; 100 µg/mL). All cells were passaged regularly every 3–4 days and were grown at 37 °C, 5% CO_2_ and 98% humidity.

### 3.2. Reagents

Anti-Flag (M2, clone 2) and β-tubulin (T 0198, clone D66) antibodies were obtained from Sigma-Aldrich (St. Louis, MO, USA). STAT5 (C-17, sc-835) antibody was from Santa Cruz Biotechnology (Dallas, TX, USA). P-STAT5 (C11C5, *9359: Tyr694 Stat5a; Tyr699 Stat5b), P-Stat3 (*9131: Tyr705) and P-STAT5-Alexa Fluor^®^647 (C71E5, *9365: Tyr694 Stat5a; Tyr699 Stat5b) antibodies were purchased from Cell Signaling (Cambridge, UK). The XTT Cell proliferation Kit II was obtained from Roche Diagnostics (Rotkreuz, Switzerland). Recombinant murine IL-3 was purchased from Sigma-Aldrich. Imatinib (Gleevec, Novartis, Basel, Switzerland) was kindly provided by Christian Wichmann (Georg-Speyer-Haus, Frankfurt am Main, Germany).

### 3.3. Plasmid Construction

The pFlag-2 vector (Sigma-Aldrich) was used for the bacterial expression of recombinant proteins. A S5-DBD-PA peptide aptamer construct was generated which allows for protein transduction, purification, nuclear translocation and immunological detection. The Stat5-DBD specific 12mer peptide aptamer sequence was integrated into the hTRXΔcys5 scaffold and a NLS-sequence, the 9-R PTD and the 6xHis tag, C-terminal of the Flag tag sequence, were added [79].

The lentiviral pLeGO-G vector (Lentiviral Gene Ontology Vectors: www.lentigo-vectors.de) was used for the stable expression of human Stat5a and Stat5b specific shRNA. The validated shRNAs were obtained from Sigma-Aldrich [MISSION^®^ shRNA], introduced into the lentiviral pLKO.1-puro vector, encoding a puromycin selectable marker gene. For the identification of transduced cells by fluorescence microscopy and for the determination of viral titers by FACS, the shRNA sequences under the control of the pLKO.1-puro U6 promoter, were cloned in the pLeGO-G vector also encoding a eGFP marker gene. The sequences of the processed siRNA antisense strands were for human Stat5a (TRCN0000019308): 5' ATCCTGATCGAGTACATGGTC 3', and for human Stat5b (TRCN0000019356): 5' ATCTGGCTTGTTAATGAGTAG 3'. A non mammalian shRNA from Sigma-Aldrich (MISSION^®^ shRNA), (*SHC002, TRC1/1.5): 5' TTGGTGCTCTTCATCTTGTTG 3', was cloned from pLKO.1-puro into the lentiviral pLeGO-C vector with mCherry red fluorescent marker.

For the stable expression of S5-DBD-PA and the hTRX scaffold control protein in target cells, the complete Flag-NLS-hTRXΔcys5-9R-6xHis cassette was cloned into the lentiviral pSiEW vector via a single SacII-restriction site. Here transgene expression was driven by a spleen focus-forming virus (SFFV) promoter, which also drives the expression of eGFP through an internal ribosome entry site (IRES). All lentiviral gene transfer vectors had an HIV-1 derived vector backbone with SIN-configuration (self-inactivating) for safety. The vectors provide for replication incompetent transgene expression, due to a deletion of promoter and enhancer sequences in the 3'-LTR.

### 3.4. Lentiviral Vector Production and Target Cell Infection

For the production of lentiviral particles, VSV-G pseudotyped lentiviral SIN-vectors were produced by calcium phosphate transfection of the pCMVΔR8.91 plasmid, encoding HIV-1 derived gag, pol, rev and tat genes, the VSV-G envelope encoding pMD2.VSV-G vector and the respective transfer plasmid into the 293T packaging cell line [83]. Cell supernatants containing the lentiviral particles were collected 48, 72, and 96 h after transfection, the viruses were concentrated by ultracentrifugation and stored in aliquots at –80 °C. The viral titer was determined by transducing 293T cells with serially diluted viral suspensions, followed by FACS analysis of the infected cells. For transduction experiments, the target cells were infected with an MOI = 20 (multiplicity of infection: amount of viral particles used for infection of one target cell). After 24 h fresh media was added to the target cells after they were washed with PBS.

### 3.5. Identification of the 12mer Peptide Aptamer (PA) Sequence, which Mediates the Interaction of the S5-DBD-PA Construct with Stat5

The 12mer PA sequence of the S5-DBD-PA construct was isolated in a yeast-two-hybrid screen. A high affinity interaction of the PA sequence with the DNA-binding domain of human Stat5a was detected [44].

### 3.6. Recombinant Expression, Protein Purification and Transduction

Recombinant expression and the subsequent purification of S5-DBD-PA and hTRX by Ni^2+^-chelate affinity chromatography, using a FPLC system, was done as described earlier [45]. For protein transduction of eukaryotic target cells, the recombinant proteins were added to cell culture media in concentrations indicated in the figure legends. To remove untransduced membrane bound proteins, the cells were washed twice with PBS and once with PBS containing 0.2 mM acetic acid prior to cell lysis (acid-wash).

### 3.7. Western Blot Analysis

Cells were washed twice with ice-cold PBS and, to remove peptides from the cell surface, additionally with acid (0.2 mM acidic acid) on ice, solubilized in radioimmunoprecipitation assay lysis buffer (50 mmol/L Tris (pH 7.4), 150 mmol/L NaCl, 1% NP40, 0.5% sodium desoxycholate, 1 mmol/L EDTA, protease inhibitiors), and incubated on ice for 20 min. Lysates were clarified by centrifugation at 16,000 × g for 10 min. For SDS-PAGE, 20–30 µg of each protein sample were loaded per lane. Gels were blotted onto nitrocellulose membranes, which were probed with specific antibodies as indicated in the figure legends. Proteins were visualized with peroxidase-coupled secondary antibodies with the chemoluminescence system (GE-Healthcare, Chalfont St Giles, UK).

### 3.8. Cell Viability and Growth Measurements: XTT-Assay, Cumulative Cell Number (CCN) Determination and annexin V/7-AAD Apoptosis Staining

XTT assays were carried out to determine cell viability and proliferation upon Stat5 inhibition. With lentivirus transduced cells, the experiment was started 24 h after infection by seeding 2,000 cells in 100 µL Medium in 96 wells. At intervals of 3 to 4 days the cells were analyzed over a period of 20 days, the medium was exchanged regularly. S5-DBD-PA protein transduction experiments were done over a period of 5 days. After seeding 2,000 cells into 96 wells, the experiments were started the following day. Fresh medium was added to the cells, containing either S5-DBD-PA (0.5; 1; 1.5 µM), hTRX (1.5 µM) or protein dialysis buffer or PBS. Protein addition and XTT-measurements were carried out in daily intervals. XTT values were measured, according to the manufacturer’s protocol (Roche Diagnostics). This assesses cell viability via bioreduction of a tetrazolium compound. Spectrophotometric quantification was done at 490 nm on a plate reader.

For detailed cell growth analyses, the cumulative cell numbers were determined. Cells were seeded at 1 × 10^5^ cells in 5 mL culture medium. In intervals of 3 to 4 days, the cells were passaged and cell numbers were counted with a hemocytometer. At every passaging, 1 × 10^5^ cells of each sample were put into 5 mL culture and the corresponding dilution factor was noted. At the final day of analysis the cumulative cell number was determined as follows: CCNi = N0 × V1 × V2 × V3 × … × Vi (N = cell number seeded; V = dilution factor of each passage).

Apoptotic cell death was detected by flow cytometry using Annexin V/7-AAD staining. Cells were harvested and 1 × 10^6^ cells were resuspended in 1ml assay buffer (10 mM HEPES, 140 mM NaCl, 2.5 mM CaCl_2_) supplemented with 2 µL Annexin V-APC (BD Biosciences, Franklin Lakes, NJ, USA) and 1 µL 7-AAD staining solutions (BD Biosciences). After 15 to 20 min incubation at RT, stained cells were analyzed by flow cytometry. Double-negative cells were considered as viable, Annexin V^+^ as early apoptotic, double-positive as apoptotic and 7-AAD^+^ as late apoptotic/necrotic cells. The results were confirmed based on changes in forward light scattering properties of dead cells, which showed decreased cell size. All fluorescence-activated cell sorting (FACS) analyses, including further eGFP-expression analyses and cell sort experiments, were carried out with FACSCanto II, FACSAria II and FACSCalibur cytometers (BD Biosciences, Heidelberg, Germany) equipped with CellQuest Pro 5.2 or FACSDiva V6.1.2 software (BD Biosciences).

### 3.9. qRT-PCR Analysis

Total cellular RNA was extracted with the NucleoSpin RNA II kit from Macherey-Nagel (Düren, Germany) and reverse transcribed with the SuperScript III—Reverse Transkriptase Kit from Invitrogen (Carlsbad, CA, USA). Amplification and quantification of cDNA was carried out with MaximaTM SYBR green qPCR Master Mix from Fermentas (St. Leon-Rot, Germany) according to the manufacturer’s protocol. qRT-PCR measurement was carried out in a Light Cycler 480 II system from Roche Diagnostics with primer sequences specific for selected Stat5 target genes. Relative quantification was done in relation to HPRT1-housekeeping gene expression and data were expressed as fold of control treated cells.

### 3.10. Immunofluorescence Staining

Lentiviral transduced and S5-DBD-PA and hTRX expressing K562 cells were grown on coverslips. Cells were fixed and permeabilized with the Cytofix/CytopermTM permeabilization and wash buffer system from BD Biosciences. For the detection of flag tagged proteins and Stat5 the cells were incubated with anti-Flag M2 primary antibody (mouse, Sigma-Aldrich) and a STAT5 primary antibody (rabbit, C-17, Santa Cruz Biotechnology) for 30 min at room temperature with Cytofix/Cytoperm wash buffer (antibody amounts were used according to the manufacturer). After multiple washing steps, an Alexa Fluor^®^546 conjugated anti-mouse secondary antibody (Life Technologies, Invitrogen) and an Alexa Fluor^®^647 conjugated anti-rabbit secondary antibody (Life Technologies, Invitrogen) was added together with vital cell staining eFluor®780 fixable viability (eBiosciences, San Diego, CA, USA) to fresh wash buffer and cells were incubated for another 30 minutes. Finally cells were prepared for confocal laser scanning microscopy by adding mounting medium (ProLong^®^ Gold Antifade; Invitrogen) and subsequently analyzed.

## 4. Conclusions

Stat3 and Stat5 activities have been found to play essential roles in solid tumors and leukemias [3,37,84,85]. Although Jak-Stat signaling is crucial for cellular functions in the mammary gland, lymphocytes, adipocytes, neuronal cells, cardiomyocytes, hepatocytes, eye cells and adult stem cells [2], the deregulation of the extent and the duration of Stat signaling convert the essential cellular signaling components into oncogenes. If strong activation of Stat3 and Stat5 is being maintained over long periods of time, signaling is associated with cellular proliferation and transformation [6].

The transcription factors Stat3 and Stat5 can both act as oncogenes under particular conditions, establish complex signaling interconnections [86] and play overlapping roles in the transactivation of target genes. However, they can assume similar and distinct functions in the process of tumorigenesis [3]. Stat3 has been found to be persistently activated in e.g., breast, prostate ovary and pancreatic tumors [87,88] and Stat5 in e.g., prostate and breast cancer [84,89], but also in various myeloproliferative diseases [28,37,39,90].

Functional domains and secondary modifications important for the transforming potential of Stat5 have been investigated. The amino terminal domain of Stat5 was found to be crucial for the induction of survival signals and the maintenance of malignant cells in myeloproliferative diseases [74]. This domain also mediates the interaction with PIAS3, a negative regulator of Stat5. PIAS3 normally inhibits the transactivation function of Stat5, an effect which is circumvented by the truncation of the amino terminal domain in prostate cancer cells [91]. In addition to the phosphorylation at tyrosine 694, the phosphorylation of the serine residues 725 and 779 have been found essential prerequisites for hematopoietic transformation mediated by Stat5 [92,93].

Stat5 is present in the cytoplasm and in the nucleus of cells and the subcellular location is dependent upon dynamic trafficking. The transport of Stat5 into the nucleus depends on an unconventional nuclear localization signal that functions within the conformation of an extensive coiled-coil domain. Its nuclear import is mediated by the importin-α3/β1 system and independent of the Stat5 activation state. Stat5 continually shuttles in and out of the nucleus mediated via chromosome region maintenance 1 (Crm1)-dependent and Crm1-independent pathways [94].

Stat proteins were discovered as phosphorylation induced transcription factors and their functions were initially thought to be restricted to the regulation of nuclear gene expression. Sophisticated localization techniques and inhibition experiments have shown that Stat proteins can assume additional functions in subcellular organelles other than the nucleus. Non-genomic functions were discovered for Stat3, it resides in the mitochondria of cells where phosphorylation at S727 is required for optimal electron transport chain activity and protection against stress-induced mitochondrial dysfunction [95]. Additional functions were also assigned to Stat5 and Stat6. Stat5 is constitutively associated with the endoplasmic reticulum and the Golgi apparatus in human pulmonary arterial endothelial cells. Stat5 depletion caused cystic changes in the endoplasmic reticulum, Golgi fragmentation and mitochondrial fragmentation. Stat6 is also found in mitochondria [58,96].

Additonal roles of Stat5, distinct from transcriptional transactivation, were also found in the nuclear compartment. Since Stat5 shuttles in and out of the nucleus, irrespective of its phosphorylation status, unphosphorylated Stat5 is present in the nucleus. Nuclear, unphosphorylated Stat5 can assume the role of a tumor suppressor. It can repress the expression of oncogenes by promoting heterochromatin formation and its stabilization through its interaction with heterochromatin protein 1 [97].

Our experiments show that Stat5 can be localized in the cytoplasm and the nucleus of K562 cells, but we found that Stat5 is nearly exclusively present in the cytoplasm of HEL cells. Mechanisms which could cause such a subcellular distribution have recently been addressed. It was found that the activities of src family kinases can cause the cytoplasmic retention of Stat5 through the interaction of the SH2 domain [72].

**Figure 9:**
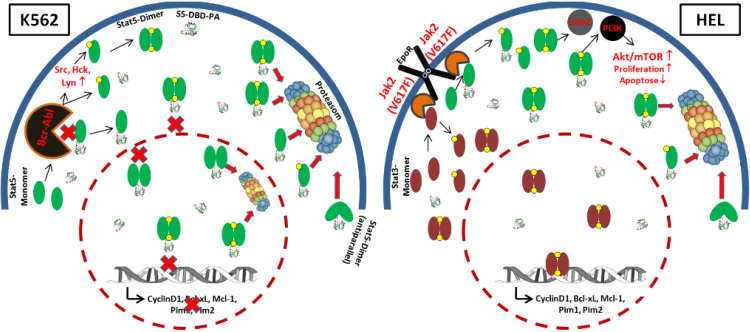
Model for the Stat5-regulated survival mechanisms in Bcr-Abl-transformed K562 and Jak2(V617F)-transformed HEL cells and the inhibitory functions of S5-DBD-PA of cytoplasmic and nuclear Stat5. Oncogenic Bcr-Abl causes the phosphorylation of Stat5 in the K562 CML cells. This can happen directly or indirectly via the activation of Src family kinases: Src, Hck, Lyn. Activated dimers of Stat5 subsequently translocate to the nucleus and drive the expression of growth promoting and antiapoptotic target genes. The interaction of S5-DBD-PA with the DBD of Stat5 interferes with DNA-binding and transcription, blocks the nuclear im- and export of active dimers and reduces tyrosine phosphorylation (phosphate groups are indicated by a yellow point) potentially through steric hindrance of complex formation between the enzyme and the substrate. It possibly causes an enhanced degradation of Stat5. The erythropoietin receptor (Epo-R) associated activity of mutant Jak2(V617F) is characteristic of Epo-hypersensitive PV and acute erythroid leukemia diseases [36]. In the HEL cell line, Stat5 and Stat3 monomers are phosphorylated by oncogenic Jak2(V617F)^+^ independent of a S5-DBD-PA interaction. Active Stat5-dimer remain in the cytoplasm and promote essential PI3K-mediated survival pathways through cofactor interactions. The lack of nuclear Stat5 activity in HEL cells is accompanied by Stat3 activation and leads to a relatively higher resistance towards S5-DBD-PA when compared to K562 cells.

The Stat5 downregulation experiments and the S5-DBD-PA mediated Stat5 inhibitor studies yielded insight into the functional importance of Stat5 present in these subcellular compartments. Our experiments showed that S5-DBD-PA is an effective Stat5 inhibitor. In addition, they showed that the survival and growth of these leukemic cell lines are not only dependent on the regulation of gene expression exerted by Stat5 in the nuclear compartment of K562 cells, but also on the cytoplasmic functions evident in HEL cells. These cell lines are dependent on two distinct Stat5 mediated survival mechanisms. A model for these mechanisms is suggested in Figure 9.

Targeting the activity of Stat5 in cancer cells has become a widely appreciated and pursued topic in drug development. Different mechanistic approaches have been applied, including the inhibition of Stat5 activating kinases [98], the interference with Stat5 binding cofactors [29] or the development of compounds which directly interact with Stat5 [99]. S5-DBD-PA is a molecule which also directly interacts with Stat5 and inhibits its functions in the cytoplasm and in the nucleus. Since this protein-interaction is dependent upon complementary interfaces, it is probably not prone to the development of resistance, a problem which limits the useful period of most targeted drugs [71].

**Figure 10:**
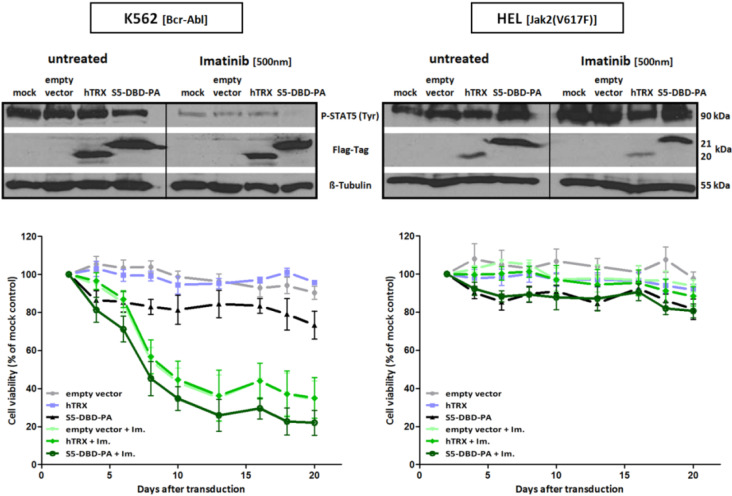
Combined imatinib and S5-DBD-PA treatment synergize in death induction of Bcr-Abl^+^ K562 cells. 2 days after the lentiviral transduction of S5-DBD-PA, the hTRX scaffold or empty vector (SiEW) K562 cells were cultured without or with imatinib (f.c.: 500 nM). Protein construct expression and the influence of combined S5-DBD-PA and imatinib treatment on P-Stat5 protein levels were analyzed by western blotting with Flag-tag and P-Stat5 specific antibodies. The lysates were prepared after 5 days of imatinib treatment. Cell growth and viability were assayed by XTT conversion from the start of imatinib treatment (day 2 after infection) till day 20 after infection (*n* = 3; Ø ± SD). The incubation of the HEL cell line served as a control of Bcr-Abl specific inhibition through imatinib.

The combination of drugs which are directed against tyrosine kinases with drugs targeting Stat5 are a promising therapeutic option [40]. We could demonstrate this by combining imatinib and S5-DBD-PA and observed drug synergisms (Figure 10). This might be based on the observation that the survival of CML stem cells, responsible for residual disease in patients, is not dependent on the activity of Bcr-Abl [100,101]. Poor pharmacokinetic properties still represents a major obstacle in the development of clinically applicable protein therapeutics. The use of modern viral or non-invasive tumor-directed cargo and transfer techniques can solve this problem in the future. Furthermore PA-interaction interfaces will gain pharmacological relevance by using them as screening platforms for the identification and design of low molecular weight therapeutics [102].

## Supplementary Files

Supplementary File 1
