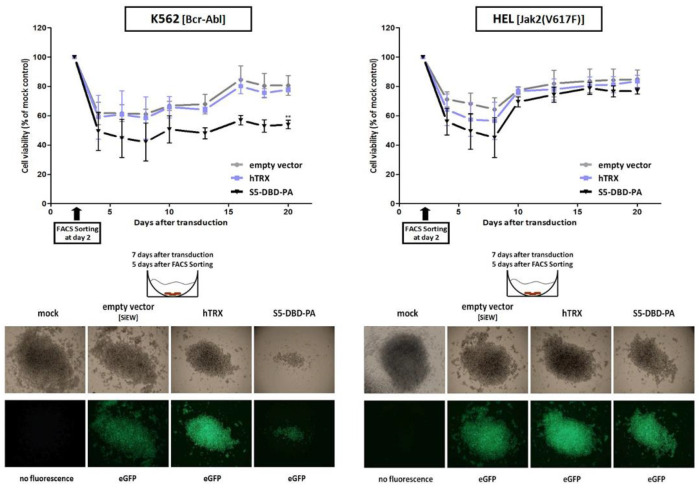# Correction: Weber et al. Stat5 Exerts Distinct, Vital Functions in the Cytoplasm and Nucleus of Bcr-Abl^+^ K562 and Jak2(V617F)^+^ HEL Leukemia Cells. *Cancers* 2015, *7*, 503–537

**DOI:** 10.3390/cancers17111754

**Published:** 2025-05-23

**Authors:** Axel Weber, Corina Borghouts, Christian Brendel, Richard Moriggl, Natalia Delis, Boris Brill, Vida Vafaizadeh, Bernd Groner

**Affiliations:** 1 Georg-Speyer-Haus, Institute for Tumor Biology and Experimental Therapy, 60596 Frankfurt am Main, Germany; axel.weber01@t-online.de (A.W.); delis@gsh.uni-frankfurt.de (N.D.); brill@gsh.uni-frankfurt.de (B.B.); vida.vafaizadeh@unibas.ch (V.V.); 2 Ganymed Pharmaceuticals AG, 55131 Mainz, Germany; c.heinz@ganymed.ag; 3 Boston Children’s Hospital, Division of Hematology/Oncology, Boston, MA 02115, USA; christian.brendel@childrens.harvard.edu; 4 Ludwig Boltzmann Institute for Cancer Research (LBI-CR), 1090 Vienna, Austria; richard.moriggl@lbicr.lbg.ac.at

## Error in Figure 8d

In the original publication [1], there was a mistake in Figure 8d as published. The image of the mock control in the case of the HEL cell line was not correct for inexplicable reasons. The corrected Figure 8d appears below. The authors state that the scientific conclusions are unaffected. This correction was approved by the Academic Editor. The original publication has also been updated.