# Supplementary material for: Stat5 Exerts Distinct, Vital Functions in the Cytoplasm and Nucleus of Bcr-Abl+ K562 and Jak2(V617F)+ HEL Leukemia Cells
Source: Cancers (Basel). 2015 Mar 19;7(1):503–37. doi: 10.3390/cancers7010503 (PMC4381271; doi:10.3390/cancers7010503)
Supplement: Supplementary File 1 [file cancers-07-00503-s001.pdf]

Supplementary Materials

a

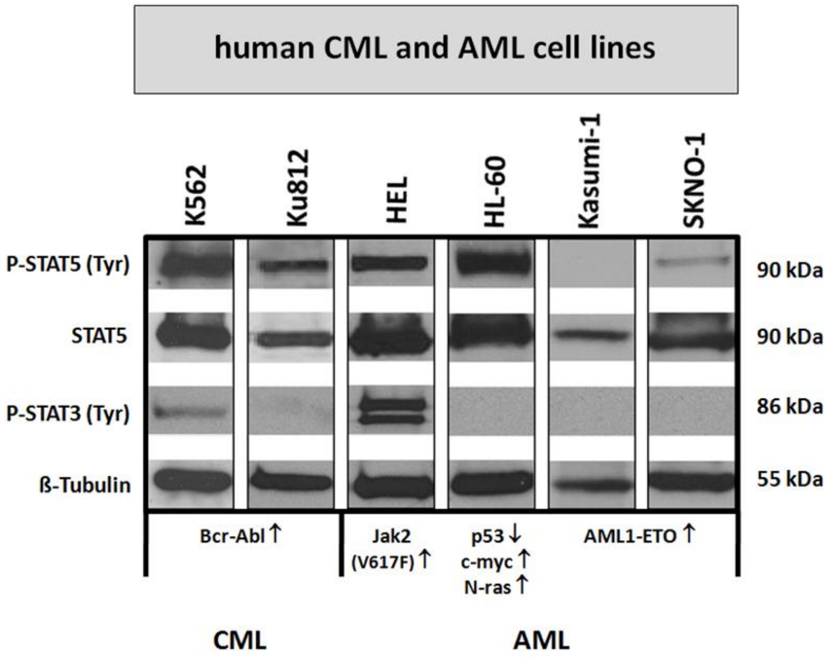

b

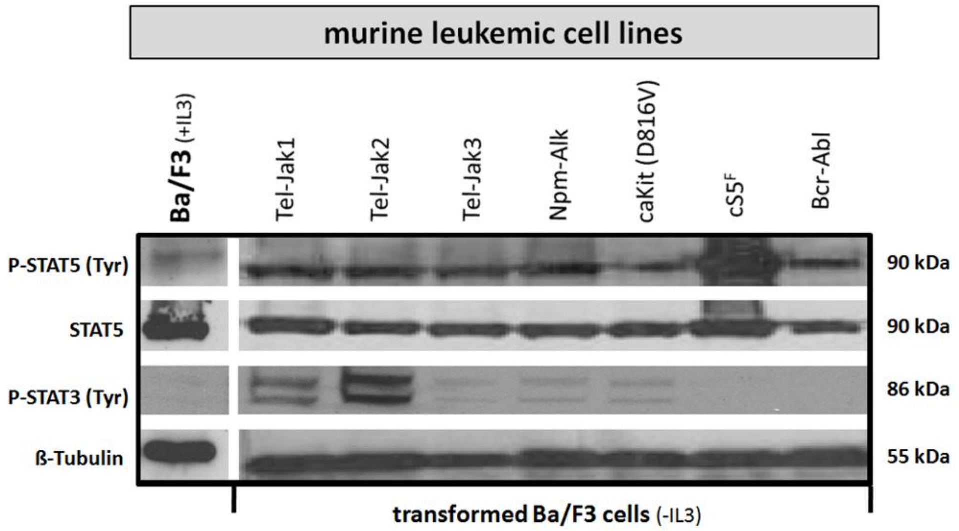

Figure S1. Stat5 expression and activation in leukemia derived cell lines. (**a**) Stat5 expression and activation in human Bcr-Abl$^+$ CML (K562, Ku812) and human Bcr-Abl$^-$ negative AML (HEL, HL60, Kasumi-1, SKNO-1) cell lines. Analysis was done on cell lysates by western blotting. Mutations underlying the transformation of these cell lines are indicated. 20 µg of protein were loaded per gel lane and analyzed with Stat5 specific antibodies recognizing both Stat5 proteins (Stat5a + Stat5b) or the activated, tyrosine phosphorylated Stat5 (Stat5a[Y694] + Stat5b[Y699]). The Stat3 activation status was determined with an antibody specific for Stat3-P-Y705. (**b**) The Western blotting analyses were done with lysates of the B-cell progenitor cell line Ba/F3, which grows IL-3-dependently. Furthermore, transformed cell variants were used, which grow IL-3-independently due to the expression of the fusion kinases Bcr-Abl, Tel-Jak and Npm-Alk or the constitutively active Stat5 (S710F) or Kit-receptor (caKit, D816V) oncogenes.

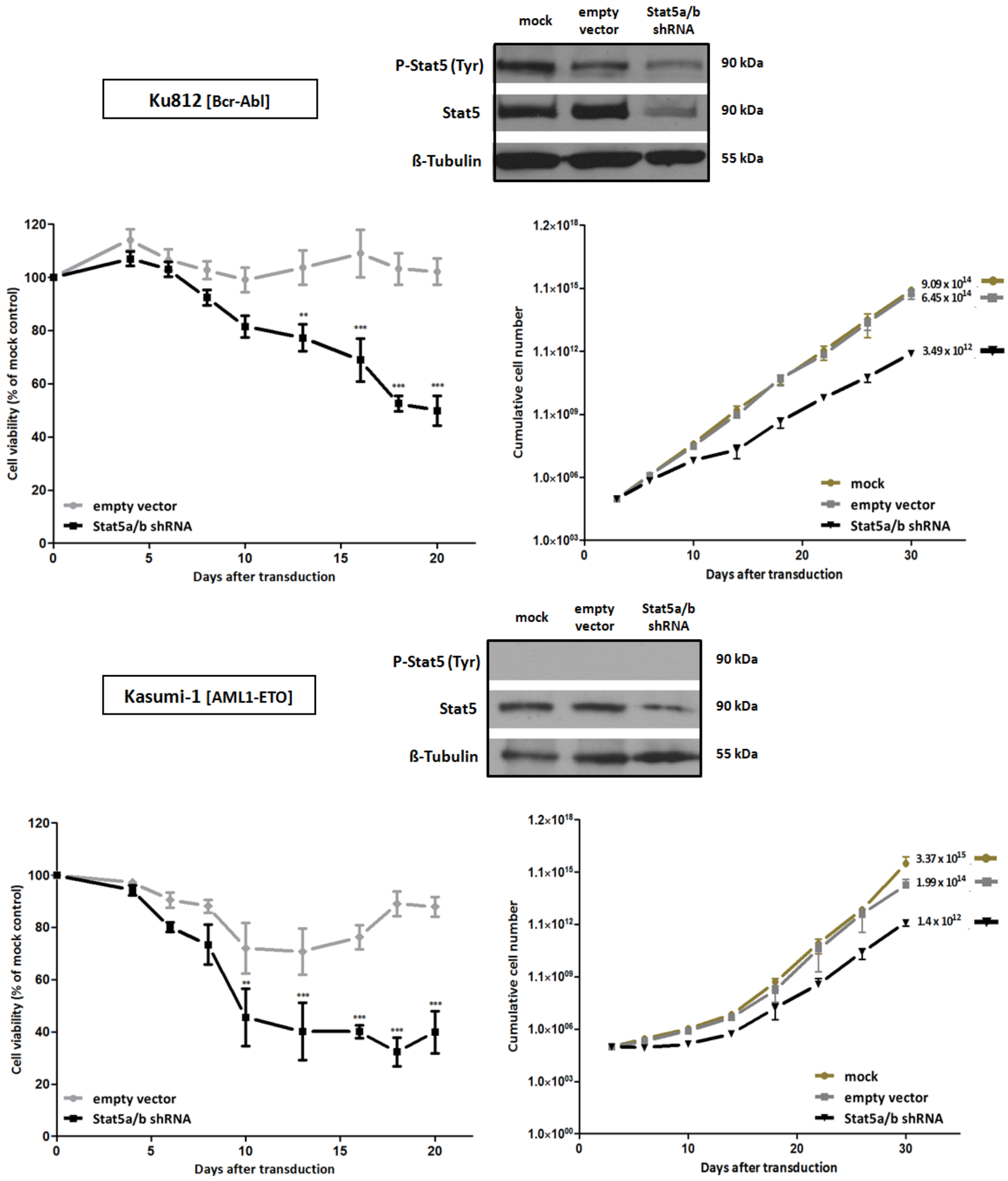

Figure S2. Knockdown of Stat5 expression by specific shRNA inhibits growth and viability of Bcr-Abl$^+$ Ku812 (CML) and AML1-ETO$^+$ Kasumi-1 (AML-M2) cells. Ku812 and Kasumi-1 cells were infected with lentiviruses encoding shRNA directed against human Stat5a and Stat5b isoforms or empty vector. Lysates were prepared after 10 days and the downregulation efficiencies were measured on the protein level with antibodies recognizing total and tyrosine phosphorylated Stat5. For proliferation and viability measurements XTT-values and the cumulative cell number were determined at regular intervals over a period of 20 to 30 days (n = 3; Ø ±SD). Significantly reduced XTT-values (percentage of mock control) in comparison to empty vector expressing cells are indicated. ** $p < 0.01$, *** $p < 0.001$ (2-way-ANOVA with Bonferroni correction).

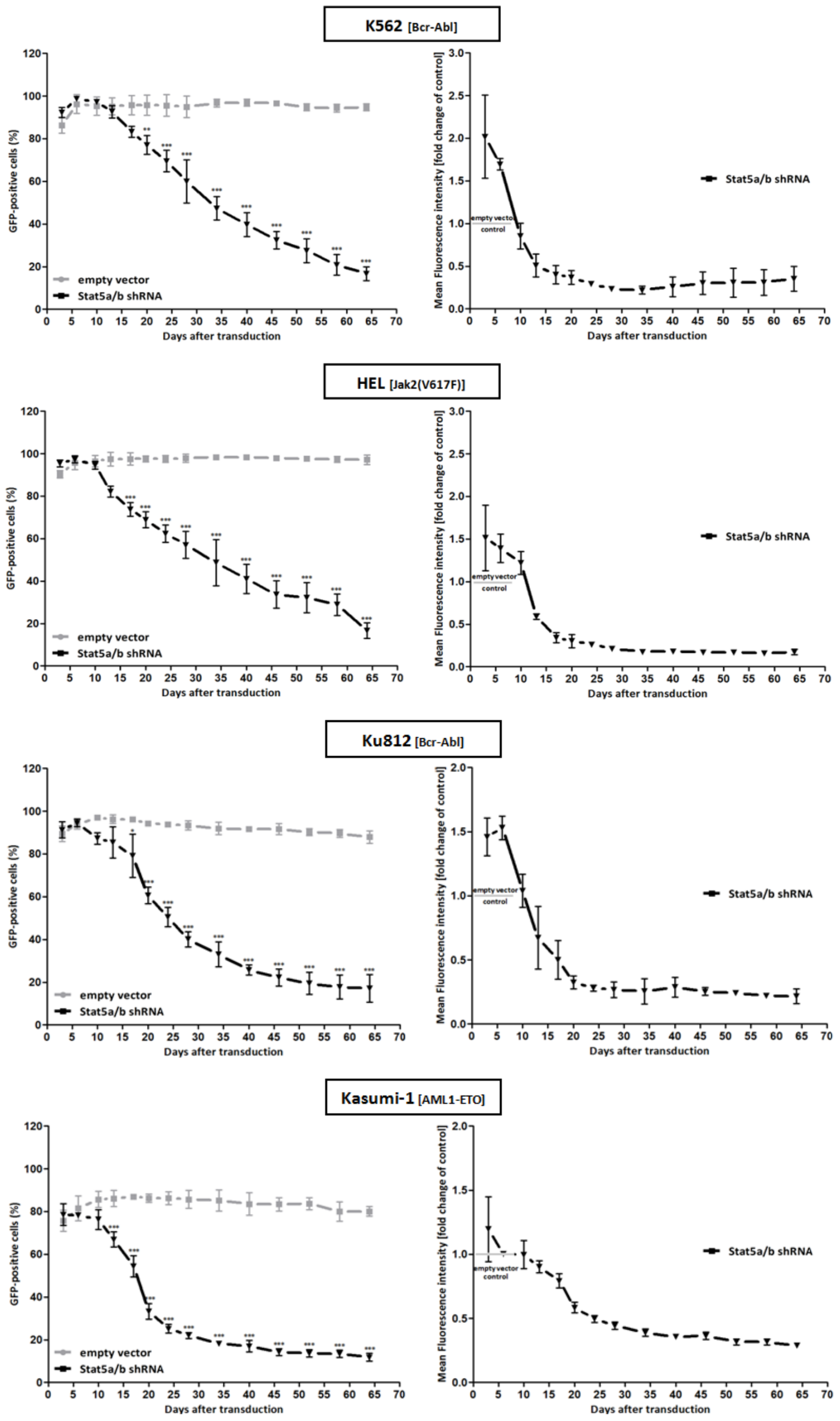

Figure S3. Measurement of the eGFP fluorescent marker gene expression in leukemic cell lines after lentiviral transduction of Stat5-specific shRNA. Over a period of 9 weeks, infected Bcr-Abl$^+$ K562 and Ku812, Jak2(V617F)$^+$ HEL and AML1-ETO$^+$ Kasumi-1 cell populations were analyzed in regular intervals and the numbers of eGFP-positive cells (%) were counted and the intensity of eGFP expression (MFI: mean fluorescence intensity) were measured by FACS (n = 3; Ø ± SD). Significantly reduced numbers of eGFP-positive cells were found when compared to empty vector expressing cells. ** $p < 0.01$, *** $p < 0.001$ (2-way-ANOVA with Bonferroni correction). The mean eGFP fluorescence intensity of shRNA expressing cells is shown as relative MFI-values (folds of empty vector control).

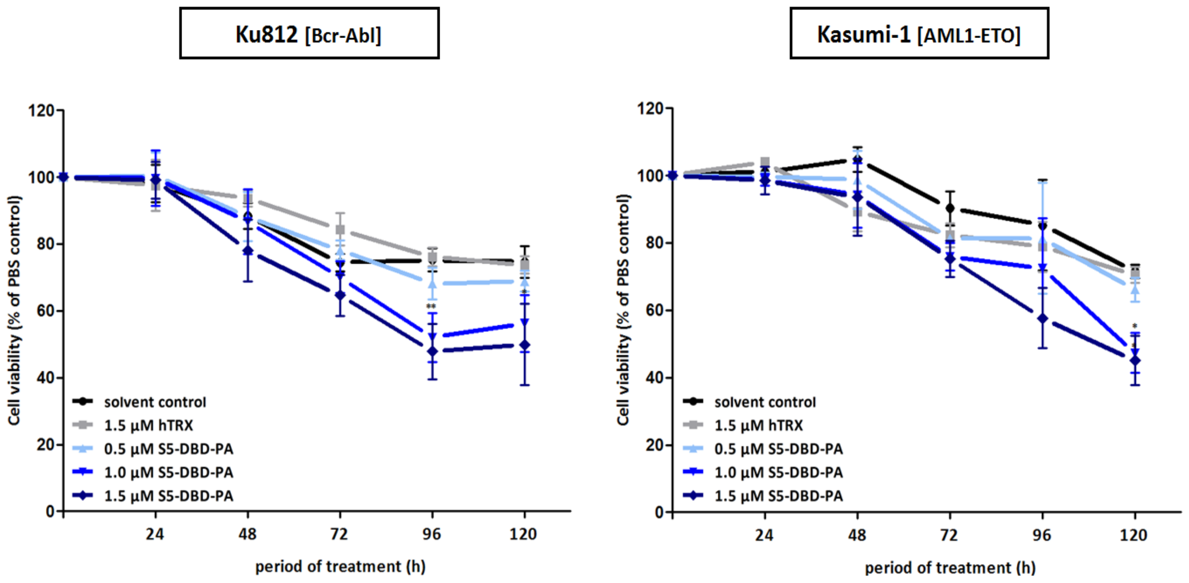

Figure S4. Inhibition of Ku812 and Kasumi-1 cell growth and viability by transduction of recombinant S5-DBD-PA. S5-DBD-PA was transduced into Bcr-Abl$^+$ Ku812 and AML1-ETO$^+$ Kasumi-1 cells by adding 0.5, 1, 1.5 µM of recombinant S5-DBD-PA to the culture medium. The treatment with the scaffold control construct (hTRX) in a 1.5 µM concentration as well as with the same volume of protein-solvent (dialysis buffer) and PBS served as negative controls. After replenishing the recombinant protein in daily intervals, cell viability and growth was monitored by XTT-measurement over a period of 5 consecutive days. Results are shown as the percentage of viable cells compared to the PBS control (n = 3; Ø ± SD). Significantly reduced XTT-values in comparison to protein solvent treated cells are indicated. * $p < 0.05$, ** $p < 0.01$ (2-way-ANOVA with Bonferroni correction).

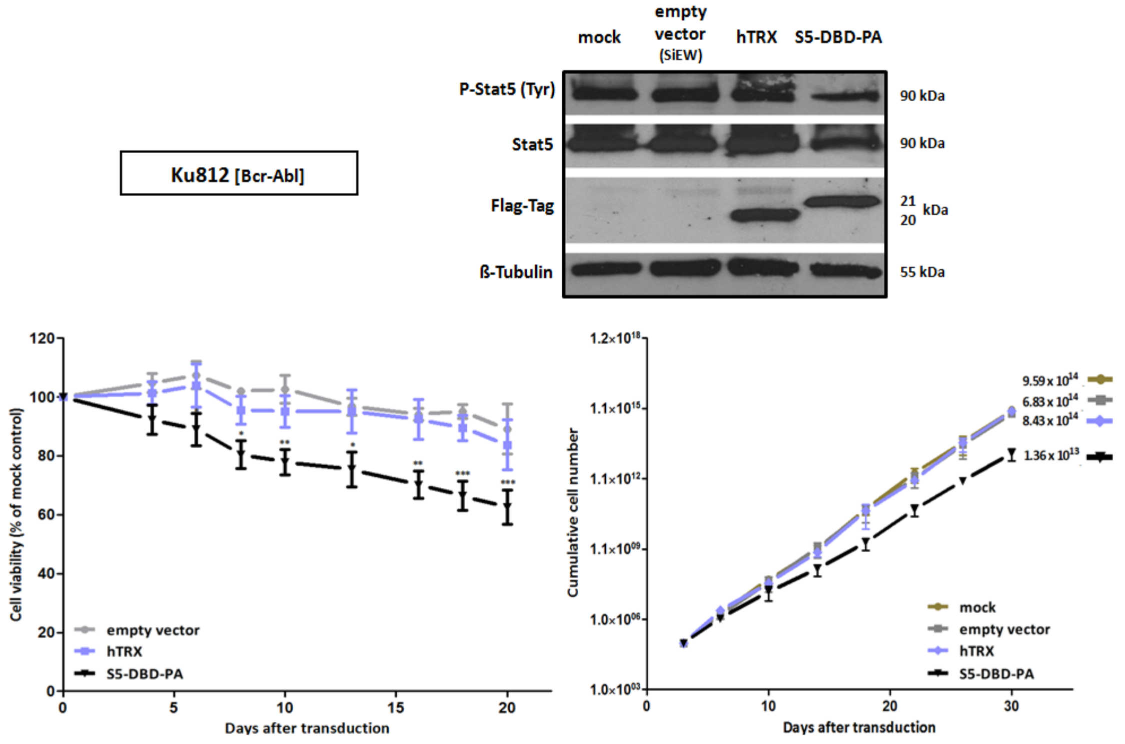

Figure S5. *Cont.*

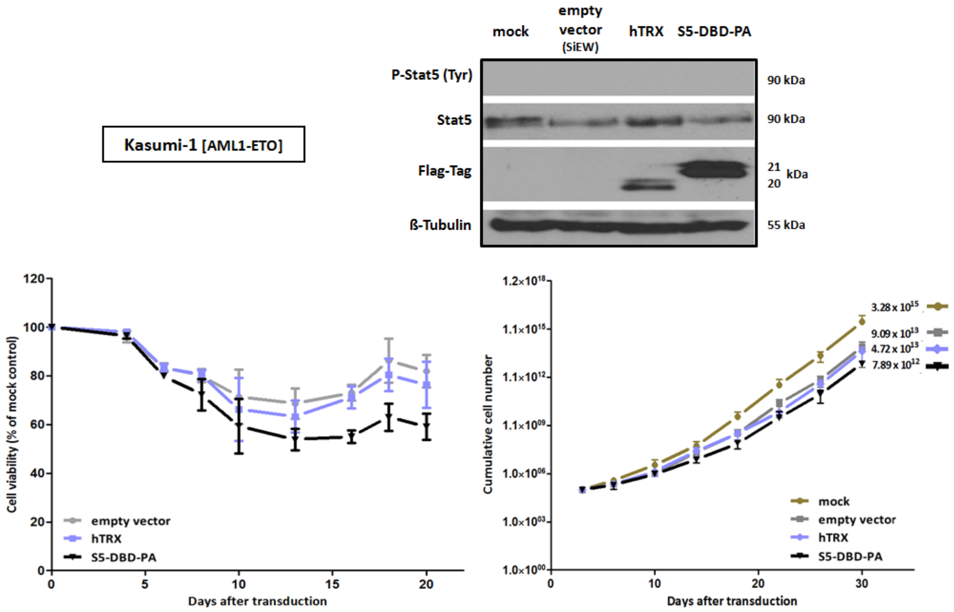

Figure S5. The endogenous expression of S5-DBD-PA differentially affects the viability of Bcr-Abl+ Ku812 and AML1-ETO+ Kasumi-1 cells. Lentiviral vectors encoding either S5-DBD-PA, hTRX scaffold or empty vector control (SiEW) were transduced into Ku812 and Kasumi-1 target cells. After 7 days, the expression of S5-DBD-PA, total and P-Stat5 were analyzed by western blotting, using Flag-tag and Stat5-directed antibodies. Growth and viability was monitored by XTT conversion and by the determination of cumulative cell numbers at regular cell passaging intervals (n = 3; Ø ± SD). Graphs indicate significantly reduced XTT-values (percentage of mock control) in comparison to empty vector expressing cells. * $p < 0.05$, ** $p < 0.01$, *** $p < 0.001$ (2-way-ANOVA with Bonferroni correction).

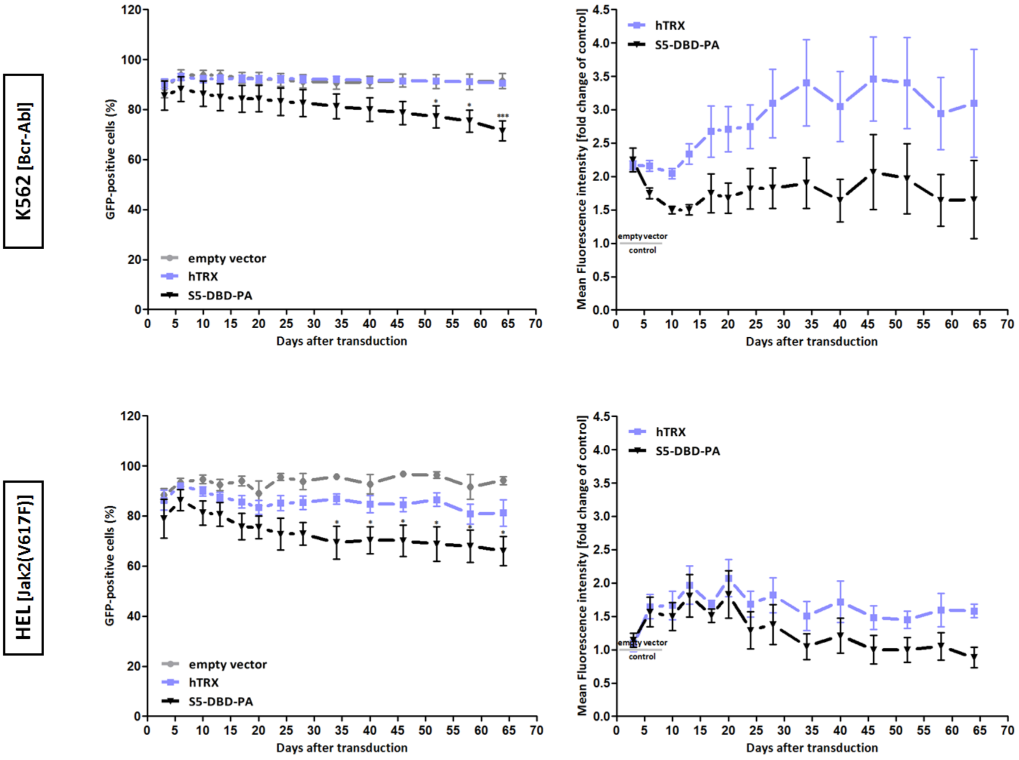

Figure S6. *Cont.*

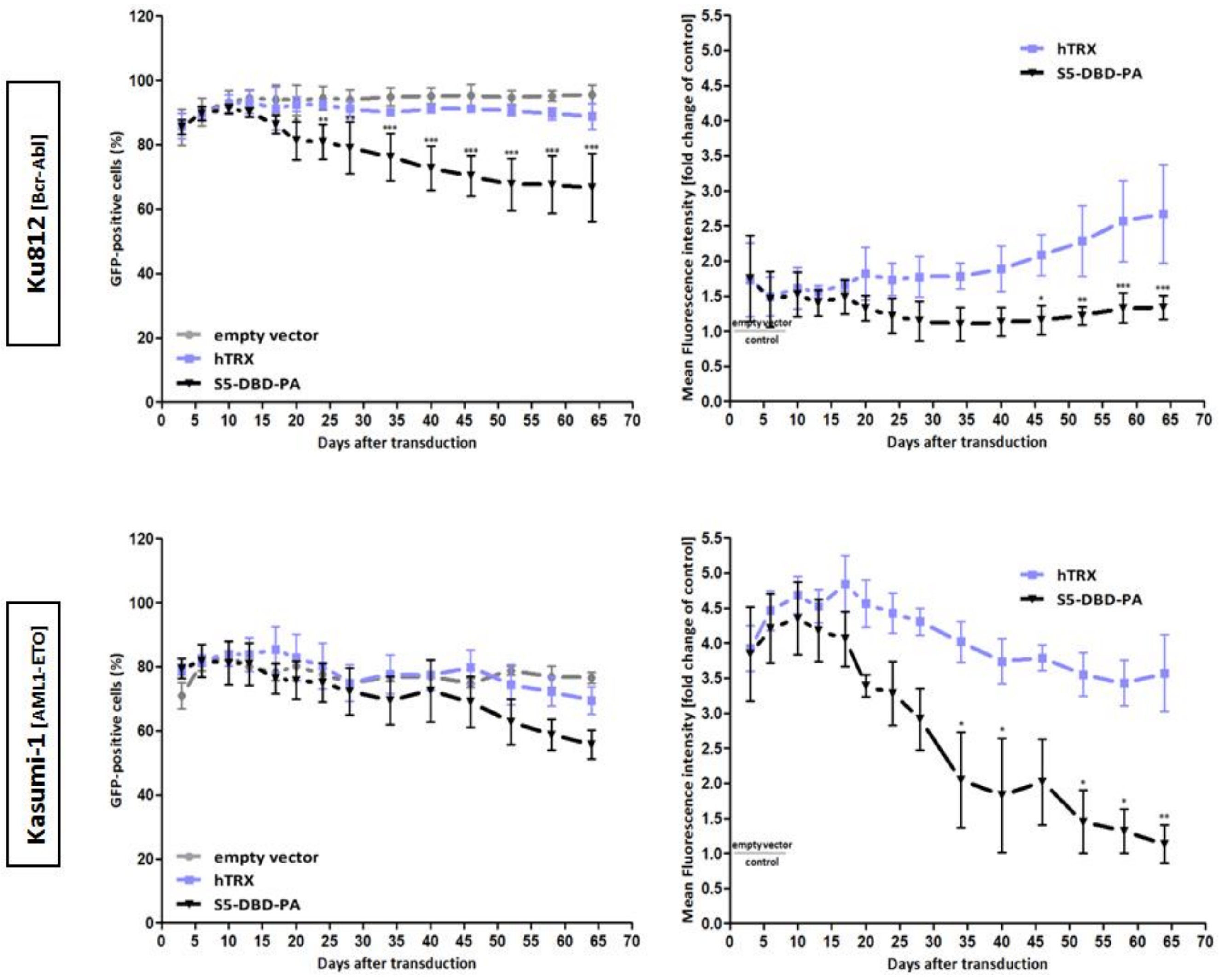

Figure S6. Reduction of eGFP-expressing leukemic cell fractions after infection with the S5-DBD-PA encoding lentivirus. The fraction of eGFP-positive K562, Ku812, HEL and Kasumi-1 cells (%) and the corresponding fluorescence intensities (MFI: mean fluorescence intensity) were measured over a period of 9 weeks by FACS (n = 6 for K562 and HEL cells, n = 3 for Ku812 and Kasumi-1 cells; Ø±SD). Graphs indicate significantly reduced numbers of eGFP-positive cells in comparison to empty vector expressing cells. * $p < 0.05$, *** $p < 0.001$ (2-way-ANOVA with Bonferroni correction). MFI-values are shown as relative folds of empty vector control. Significantly reduced MFI-values are indicated in comparison to hTRX scaffold control protein expression. * $p < 0.05$, ** $p < 0.01$, *** $p < 0.001$ (2-way-ANOVA with Bonferroni correction).
